# Resting-state EEG power differences in autism spectrum disorder: a systematic review and meta-analysis

**DOI:** 10.1038/s41398-023-02681-2

**Published:** 2023-12-14

**Authors:** Wei Siong Neo, Dan Foti, Brandon Keehn, Bridgette Kelleher

**Affiliations:** 1 Department of Psychological Sciences, Purdue University, West Lafayette, IN USA; 2 Department of Speech, Language, and Hearing Sciences, Purdue University, West Lafayette, IN USA

**Keywords:** Psychology, Autism spectrum disorders, Diagnostic markers

## Abstract

Narrative reviews have described various resting-state EEG power differences in autism across all five canonical frequency bands, with increased power for low and high frequencies and reduced power for middle frequencies. However, these differences have yet to be quantified using effect sizes and probed robustly for consistency, which are critical next steps for clinical translation. Following PRISMA guidelines, we conducted a systematic review of published and gray literature on resting-state EEG power in autism. We performed 10 meta-analyses to synthesize and quantify differences in absolute and relative resting-state delta, theta, alpha, beta, and gamma EEG power in autism. We also conducted moderator analyses to determine whether demographic characteristics, methodological details, and risk-of-bias indicators might account for heterogeneous study effect sizes. Our literature search and study selection processes yielded 41 studies involving 1,246 autistic and 1,455 neurotypical individuals. Meta-analytic models of 135 effect sizes demonstrated that autistic individuals exhibited reduced relative alpha (*g* = −0.35) and increased gamma (absolute: *g* = 0.37, relative: *g* = 1.06) power, but similar delta (absolute: *g* = 0.06, relative: *g* = 0.10), theta (absolute: *g* = −0.03, relative: *g* = −0.15), absolute alpha (*g* = −0.17), and beta (absolute: *g* = 0.01, relative: *g* = 0.08) power. Substantial heterogeneity in effect sizes was observed across all absolute (*I*^2^: 36.1–81.9%) and relative (*I*^2^: 64.6–84.4%) frequency bands. Moderator analyses revealed that age, biological sex, IQ, referencing scheme, epoch duration, and use of gold-standard autism diagnostic instruments did not moderate study effect sizes. In contrast, resting-state paradigm type (eyes-closed versus eyes-open) moderated absolute beta, relative delta, and relative alpha power effect sizes, and resting-state recording duration moderated relative alpha power effect sizes. These findings support further investigation of resting-state alpha and gamma power as potential biomarkers for autism.

## Introduction

Autism spectrum disorder (ASD) is characterized by persistent and pervasive deficits in social communication and interactions as well as restricted and repetitive patterns of behaviors, interests, or activities [1]. Although many autistic individuals live fulfilling lives, ASD diagnoses are associated with impaired quality of life and significant socioeconomic costs [2–4]. Early intervention can help reduce these psychosocial costs and ASD may be reliably diagnosed as early as 14 months of age [5]. However, epidemiological and meta-analytic studies suggest that ASD is more typically diagnosed between 43 and 50 months of age [6, 7]. This discrepancy between potential and actual ASD diagnostic ages stems from multiple clinical challenges, including long wait times between initial concerns and diagnostic evaluations, sociocultural and geographical barriers in accessing diagnostic services, resource-intensive diagnostic processes that require highly trained clinicians and behavioral observations over extended periods of time, and complex differential diagnoses at young ages [8–13]. Alternate, complementary approaches are therefore needed to facilitate earlier diagnosis of ASD, with recent work focusing on potential biomarkers and digital phenotyping that could be implemented prior to the full expression of ASD symptoms [14, 15]. Notably, a multi-tiered assessment approach that incorporates diagnostic biomarkers, which are sequentially administered as needed for positively screened cases and those with diagnostic uncertainty, has recently been shown to have the potential for increasing diagnostic efficiency and decreasing lifetime costs associated with ASD [16]. To successfully implement such approaches, it is crucial to identify multiple scalable biomarkers that effectively differentiate between autistic and neurotypical individuals. Quantifying the diagnostic properties of these biomarkers (e.g., effect size, sensitivity, and specificity) will further optimize their deployment in tiered assessment services.

Neural biomarkers hold particular promise to enhance early ASD diagnosis given ASD is theorized to be rooted in structural differences in neural systems and disruptions to neural information processing [17, 18]. Indeed, systematic reviews and meta-analyses of neuroimaging and event-related potential studies have found significant anatomical and functional differences in ASD [19–22]. Resting-state electroencephalography (EEG) may also serve as an ASD biomarker for several reasons. First, similar to other neural-based measures, resting-state EEG yields objective metrics that may be more sensitive in detecting subtle neurophysiological changes that precede behavioral manifestations of ASD. Second, EEG is more cost-effective, non-invasive, or portable than other neuroimaging methodologies. Third, resting-state EEG data may be collected from individuals with a wide range of developmental and functioning levels, including young children, which is of paramount importance for early diagnosis of ASD [23]. Further, while some autistic individuals with sensory sensitivities may find it challenging to tolerate wearing EEG caps, desensitization procedures have been successfully implemented to improve compliance in EEG data acquisition [24]. Finally, relative to event-related potential techniques, resting-state EEG paradigms are typically more accessible with fewer participation demands and technological requirements. In fact, given that clinical EEG protocols routinely use resting-state paradigms, existing healthcare infrastructure (e.g., EEG recording facilities, data processing pipelines, and specialized technicians) may be leveraged when using resting-state EEG to aid in diagnosing ASD [25].

While several metrics may be used to quantify resting-state EEG signals, power spectral analysis has been the dominant approach and can be traced back to the very first human EEG study by Hans Berger [26]. Neural oscillations are typically broken down into five canonical EEG frequency bands during power spectral analyses: delta (<4 Hz), theta (4–8 Hz), alpha (8–13 Hz), beta (13–30 Hz), and gamma (>30 Hz) bands [27]. These frequency bands have been posited to map onto various affective and cognitive processes. To illustrate, delta oscillations are prominent during processing of motivational and salient stimuli [28, 29]; theta oscillations are associated with emotion regulation and memory encoding [28–30]; alpha oscillations largely reflect regulatory processes for inhibiting task-irrelevant cortical areas [28–30]; beta oscillations are involved in attentional activation and sensorimotor integration [28]; and gamma oscillations are related to binding of perceptual features, maintenance of memory contents, and representation of objects [28, 30, 31]. Additionally, EEG power spectral analyses may be conducted for both absolute power (i.e., integral of all EEG power values within a frequency band) and relative power (i.e., the ratio of absolute power for a frequency band to total absolute power across all frequency bands). These two EEG power measures therefore offer complementary insights, with absolute power ideal for characterizing the magnitude of neural oscillations and relative power helpful for understanding relationships across frequency bands.

An emerging literature suggests that absolute and relative resting-state power may be different in ASD across all five canonical frequency bands, though findings are mixed. To our knowledge, five narrative reviews have examined resting-state EEG power in ASD and are summarized in Supplementary Table 1 [32–36]. Collectively, study findings suggest a potential U-shaped profile of resting-state EEG power differences in ASD [36]; increased EEG power is observed for low and high frequencies (i.e., delta, theta, beta, and gamma bands), whereas EEG power is reduced for middle frequencies (i.e., alpha band). The reasons for these suspected differences remain unknown but possibly relate to neurofunctional differences in gamma-aminobutryic acid (GABA) neural systems between autistic and neurotypical individuals [36]. Importantly, EEG power spectral differences in ASD also appear to track with severity of clinical ASD features. For example, lower levels of resting-state alpha power are associated with preferential attention to details [37] and resting-state gamma power and social reciprocity are significantly related in ASD [38]. Although preliminary, these studies highlight the potential value of resting-state EEG power in delineating ASD clinical phenotypes.

Despite the promise of resting-state EEG power as a potential neural biomarker for ASD, three major gaps remain. First, existing reviews are narrative in nature and no meta-analysis has been conducted. While narrative reviews are valuable for summarizing prior literature, meta-analyses offer a statistical approach to synthesize study findings by determining pooled effect sizes, resolve potential discrepancies in past studies, and provide estimates of potential biases in the literature. Importantly, meta-analyses of EEG power have been successfully conducted in other neurodevelopmental disorders (e.g., attention-deficit/hyperactivity disorder), supporting similar approaches for ASD [39].

Second, existing narrative reviews on resting-state EEG power in ASD have highlighted how varied demographic characteristics and methodological decisions across studies may have contributed to differential findings, potentially influencing interpretations and translational applications of resting-state EEG power differences. For example, ASD and intellectual disability cooccur at high rates and resting-state EEG power is known to be associated with IQ [40, 41]; it is therefore possible that resting-state EEG power differences between autistic and neurotypical individuals may be confounded by group differences in intellectual abilities. Meta-analyses are well suited to systematically examine these potential moderators and quantify their influences on the heterogeneity in study findings. Additionally, meta-analyses may identify which methodological procedures are critical for detecting resting-state EEG power differences in ASD. For example, eyes-open paradigms may be particularly useful for differentiating between autistic and neurotypical individuals in some frequency bands, whereas eyes-closed paradigms may be more optimal for other frequency bands. Understanding the impact of both demographic and methodological differences is a critical preliminary step in determining the utility of resting-state EEG power as an ASD biomarker.‘

Finally, existing reviews on resting-state EEG power in ASD have not fully complied with field-standard Preferred Reporting Items for Systematic Reviews and Meta-Analyses (PRISMA) guidelines [42]. For example, some of these reviews did not specify search sources and strategies, used a single information source, and did not consider gray literature. Further, since the publication of existing narrative reviews, multiple new studies have examined resting-state EEG power in ASD and are in need of integration with past literature bases. Thus, a comprehensive systematic review and meta-analysis that adheres to PRISMA guidelines is needed to provide an updated synthesis and quantification of resting-state EEG power differences in ASD.

This systematic review and meta-analysis has two key objectives. Our primary objective is to systematically review both published and gray literature on resting-state EEG power in ASD and synthesize effect sizes of differences in absolute and relative power between autistic and neurotypical individuals across all five canonical EEG frequency bands. Consistent with prior narrative reviews, we predicted that ASD would be broadly characterized by significantly greater EEG power (i.e., positive effect sizes) for delta, theta, beta, and gamma bands and significantly reduced EEG power (i.e., negative effect sizes) for the alpha band. Our secondary aim is to quantify heterogeneity in effect sizes across individual studies and evaluate potential sources that may account for heterogeneous study findings. We expected key demographic characteristics, methodological details, and risk-of-bias indicators to moderate effect sizes of resting-state EEG power differences in ASD.

## Methods

We conducted this systematic review and meta-analysis in accordance with PRISMA guidelines [42]. We preregistered the present study on Open Science Framework (OSF; https://osf.io/p3m9y) and adhered to our preregistered plans unless otherwise noted.

### Literature search

#### Information sources

We conducted comprehensive literature searches in APA PsycInfo (EBSCO), Cochrane Library, MEDLINE (PubMed), Scopus, and Web of Science Core Collection. To identify additional gray literature, we searched ClinicalTrials.gov and ProQuest Dissertations and Theses. Each electronic database was searched from its inception to December 31, 2021, providing a contemporary synthesis of the literature and facilitating future updating efforts. We also searched electronically available conference proceedings of the International Society for Autism Research (2004–2021), Society for Neuroscience (2006–2021), and Society for Psychophysiological Research (2001–2021). Studies cited in existing narrative reviews on resting-state EEG in ASD and psychiatric disorders were examined [32–36]. For each included study, we performed backward and forward citation searches, with the latter being conducted in both Scopus and Web of Science Core Collection citation databases.

#### Search strategies

We searched study titles, abstracts, and keywords using a combination of two search terms. The search term to identify ASD studies is (ASD OR PDD OR autis* OR “pervasive development*” OR Asperger*). The search term to identify resting-state EEG studies is ((rest* OR baseline OR oscillat* OR quantitative OR spontaneous) AND (EEG OR qEEG OR electroencephal* OR electrophysio*)). We also included index terms unique to individual electronic databases, such as “autism spectrum disorders” as a specific descriptor in APA PsycInfo and “electroencephalography” as a specific medical subject heading in MEDLINE. Full line-by-line search strategies for individual information sources are detailed in Supplementary Materials.

### Study selection

#### Eligibility criteria

To be eligible, studies identified from literature searches had to meet all of the following inclusion criteria: (1) contained original research; (2) included a sample of autistic individuals with a clinical diagnosis of ASD, based on either the Diagnostic and Statistical Manual of Mental Disorders (DSM) or the International Classification of Diseases (ICD) diagnostic classification system; (3) included a sample of neurotypical individuals; (4) recorded resting-state EEG using an eyes-closed paradigm and/or an eyes-open paradigm, with the constraint that only simple visual stimuli (i.e., fixation cross, bubbles, or geometric shapes), if any, were used; (5) compared absolute and/or relative power between autistic and neurotypical individuals for at least one canonical EEG frequency band; (6) reported sufficient statistical information to compute at least one effect size; and (7) published in English before December 31, 2021.

Additionally, we applied the following exclusion criteria. Studies that exclusively focused on syndromic ASD (e.g., fragile X syndrome) and thus did not include separate samples of individuals with idiopathic autism were excluded due to different developmental trajectories between syndromic and idiopathic ASD [43]. Studies that focused on sleep EEG were excluded due to different patterns of neural oscillations during the waking state and various sleep stages [44]. Studies that focused on EEG spectral measures that were derived from absolute or relative power (e.g., coherence, power asymmetry, and power ratio) were excluded if original EEG power data were not also reported. While not explicitly indicated in our preregistration, for clarity, studies that focused on young children with high likelihood of receiving a clinical diagnosis of ASD (e.g., infant siblings of autistic individuals) and did not report ASD outcomes data were excluded; however, in cases where ASD outcomes data were reported, findings from high-likelihood children receiving a clinical diagnosis of ASD (i.e., autistic children) and low-likelihood children not receiving a clinical diagnosis of ASD (i.e., neurotypical children) were included.

#### Selection process

Studies identified from literature searches were selected for inclusion through a four-stage process. First, we removed duplicate study records based on unique study identifiers (e.g., digital object identifiers). Second, for each study record, two coders independently screened its title and abstract against inclusion and exclusion criteria. Disagreements were resolved by discussion. Third, we downloaded full-text articles, each of which was independently screened by two coders against inclusion and exclusion criteria. Disagreements were also resolved by discussion. Finally, we assessed whether different studies used overlapping samples (e.g., conference poster subsequently published as a journal article) by juxtaposing study authors, sample characteristics, and EEG methodological details. If multiple studies used overlapping samples, we prioritized published studies and those with larger sample sizes for inclusion.

### Data collection

For each included study, two coders independently extracted study metadata, sample characteristics, EEG recording, preprocessing, and spectral analysis parameters, and EEG power metrics. Discrepancies were resolved by discussion. Definitions for individual data items are described in Supplementary Materials, with the full dataset available on OSF (https://osf.io/uk92c). Here, we specifically highlight details for data items central to our study objectives, including potential moderators and study outcomes.

#### Potential moderators

Demographic Characteristics: We focused on three key demographic characteristics known to moderate resting-state EEG power: biological sex, age, and IQ [41, 45–48]. These demographic characteristics were coded separately for the autistic and neurotypical groups. We coded biological sex as the percentage of individuals who were male. We coded age as the mean age in years. We coded IQ as the mean overall, nonverbal, and/or verbal IQ. For analytic purposes, we calculated weighted percentages and means to represent the demographic characteristics of the full sample in each included study.

Methodological Details: We examined three key study methodological differences informed by prior narrative reviews: resting-state paradigm, referencing scheme, and epoch duration [36]. We coded resting-state paradigm as either eyes-closed or eyes-open paradigm. We coded referencing scheme as the re-referencing electrode or scheme if offline re-referencing was conducted (e.g., mastoids) and as the online reference electrode or scheme if offline re-referencing was not conducted (e.g., electrode Cz). We coded epoch duration as the duration of individual epochs used for analyzing EEG power in seconds. Moreover, we investigated an additional study methodological parameter not included in our preregistration: resting-state EEG recording duration, which served as a proxy for the number of artifact-free epochs used in EEG power analyses, given that the latter had not been consistently reported in published studies. We coded recording duration as the total duration of the resting-state paradigm in minutes.

Risk-of-Bias Indicators: We assessed two potential sources of bias that might limit interpretations of resting-state EEG power differences between autistic and neurotypical groups: use of gold-standard ASD diagnostic instruments and matched group design. For use of gold-standard ASD diagnostic instruments, we coded whether studies used the Autism Diagnostic Observation Schedule, Second Edition (ADOS-2) or Autism Diagnostic Interview-Revised (ADI-R) or earlier editions to determine the presence or absence of ASD [49, 50]. For matched group design, we coded whether autistic and neurotypical groups were explicitly matched or assessed to be statistically equivalent on biological sex, age, and IQ separately.

#### Study Outcomes

We were interested in 10 study outcomes: absolute and relative power across delta, theta, alpha, beta, and gamma frequency bands. For each included study, we extracted data for all reported study outcomes. Specifically, for each study outcome, we coded frequency band limits and regions of interest. We coded frequency band limits as the frequencies used to define the lower and upper limits of the canonical frequency band in Hz (e.g., 8 and 12 Hz for alpha band). We coded all reported regions of interest, with each region of interest coded as one of the following topographical regions: global, frontal, central, parietal, temporal, or occipital. For studies that analyzed EEG power using individual electrodes, we assigned individual electrodes to regions of interest based on their topographical locations (e.g., electrode Fz assigned to frontal region). For studies that analyzed EEG power using non-predefined regions of interest, we recoded them whenever possible (e.g., left frontal region recoded to frontal region, posterior region consisting only of parietal electrodes recoded to parietal region, and left and right hemispheric regions recoded to global region); if recoding to predefined regions of interest was not possible (e.g., posterior region consisting of parietal, temporal, and occipital electrodes), we excluded them to maintain consistency across studies.

For each study outcome and region of interest, we coded descriptive or inferential statistics related to EEG power, in the following order of preference: (1) mean and standard deviation values of EEG power for both autistic and neurotypical groups; (2) effect sizes (e.g., Cohen’s *d*) of EEG power differences between the two groups; and (3) test statistics (e.g., *t*-values and *F*-values) and (4) *p*-values associated with statistical tests of EEG power differences between the two groups. We coded exact *p*-values if available. To be conservative, we assumed a *p*-value of .05 if a significance test was only reported as being statistically significant; we assumed a *p*-value of 1.00 if a significance test was only reported as being non-statistically significant, corresponding to a null effect size. For studies that did not report any of these descriptive or inferential statistics, we contacted study authors to obtain relevant data. Additionally, for studies that graphically presented EEG power differences between autistic and neurotypical groups, we adopted a conservative approach in assuming *p*-values of .05 and 1.00 for non-overlapping and overlapping confidence intervals, respectively.

### Data analytic plan

We performed data analyses in R using *metafor* [51] and *meta* [52] packages. Analysis data and scripts are available on OSF (https://osf.io/uk92c).

#### Effect measures

To quantify effect sizes of EEG power differences between autistic and neurotypical groups for each study outcome and region of interest, we computed Hedges’ *g* using the in-built *escalc* function in *metafor*. Positive (negative) effect sizes indicated greater (smaller) absolute or relative power in autistic individuals than neurotypical individuals. Following expanded rules of thumb for interpreting effect sizes [53], effect sizes of 0.01, 0.2, 0.5, 0.8, 1.2, and 2.0 were considered very small, small, medium, large, very large, and huge, respectively.

To ensure independence of effect sizes within each study outcome, we adopted the following procedures to deal with multiple effect sizes in an included study. Studies that analyzed EEG power using individual electrodes would have multiple effect sizes for a region of interest (e.g., three effect sizes for electrodes F3, Fz, and F4 that were all assigned to frontal region). For such cases, we averaged effect sizes and corresponding variances given the close spatial proximity of individual electrodes within a region of interest. Similarly, studies that analyzed EEG power using multiple regions of interest would yield multiple effect sizes (e.g., five effect sizes for frontal, central, parietal, temporal, and occipital regions of interest). For such cases, we aggregated effect sizes into a single effect size, broadly representing global EEG power differences, using the in-built *aggregate.escalc* function in *metafor*. Specifically, the variance-covariance matrix of sampling errors was assumed to have a compound symmetric structure with a medium-sized correlation of .3.^1^

#### Study synthesis

We conducted 10 primary study syntheses that corresponded to the 10 study outcomes (i.e., absolute delta, theta, alpha, beta, and gamma power, and relative delta, theta, alpha, beta, and gamma power). For each study synthesis, we conducted preliminary analyses to identify outlier effect sizes; this step was not specified in our preregistration but was determined to be necessary for accurately synthesizing prior study findings. Specifically, studies with externally standardized residuals that exceeded ±1.96 were considered outliers and excluded from subsequent analyses [54]. Thereafter, we used a forest plot to visually depict and tabulate individual and pooled study effect sizes and confidence intervals. We also performed a meta-analysis by fitting a random-effects model with inverse-variance weights, given that effect sizes were likely to vary across studies due to phenotypic heterogeneity in ASD as well as differences in sample characteristics and EEG methodological decisions. We used the restricted maximum likelihood estimator, which provides approximately unbiased estimates of between-study heterogeneity variance [55]. We used the Hartung-Knapp-Sidik-Jonkman method for inferential tests of model coefficients and confidence intervals, which applies an adjustment to standard errors of estimated coefficients to account for uncertainty in residual heterogeneity estimates [56, 57]. To statistically evaluate between-study heterogeneity, we used Cochran’s *Q* tests of heterogeneity [58], with *p*-values less than .05 indicative of heterogeneity. We further quantified between-study heterogeneity using Higgins and Thompson’s *I*^2^ [59], with values of 25%, 50%, and 75% representing low, moderate, and high heterogeneity, respectively [60].

We explored possible causes of between-study heterogeneity by conducting moderator analyses for primary meta-analytic models with at least moderate heterogeneity. For categorical moderators, we conducted subgroup analyses with dummy coding. For continuous moderators, we conducted meta-regression analyses. Specifically, for each moderator, we extended the primary meta-analytic model by including the moderator, resulting in a mixed-effects model. To ensure reasonable statistical power, we restricted subgroup analyses by including only subgroups with at least five studies; similarly, we restricted meta-regression analyses by requiring at least five studies [61].

## Results

### Search and selection results

**Fig. 1:**
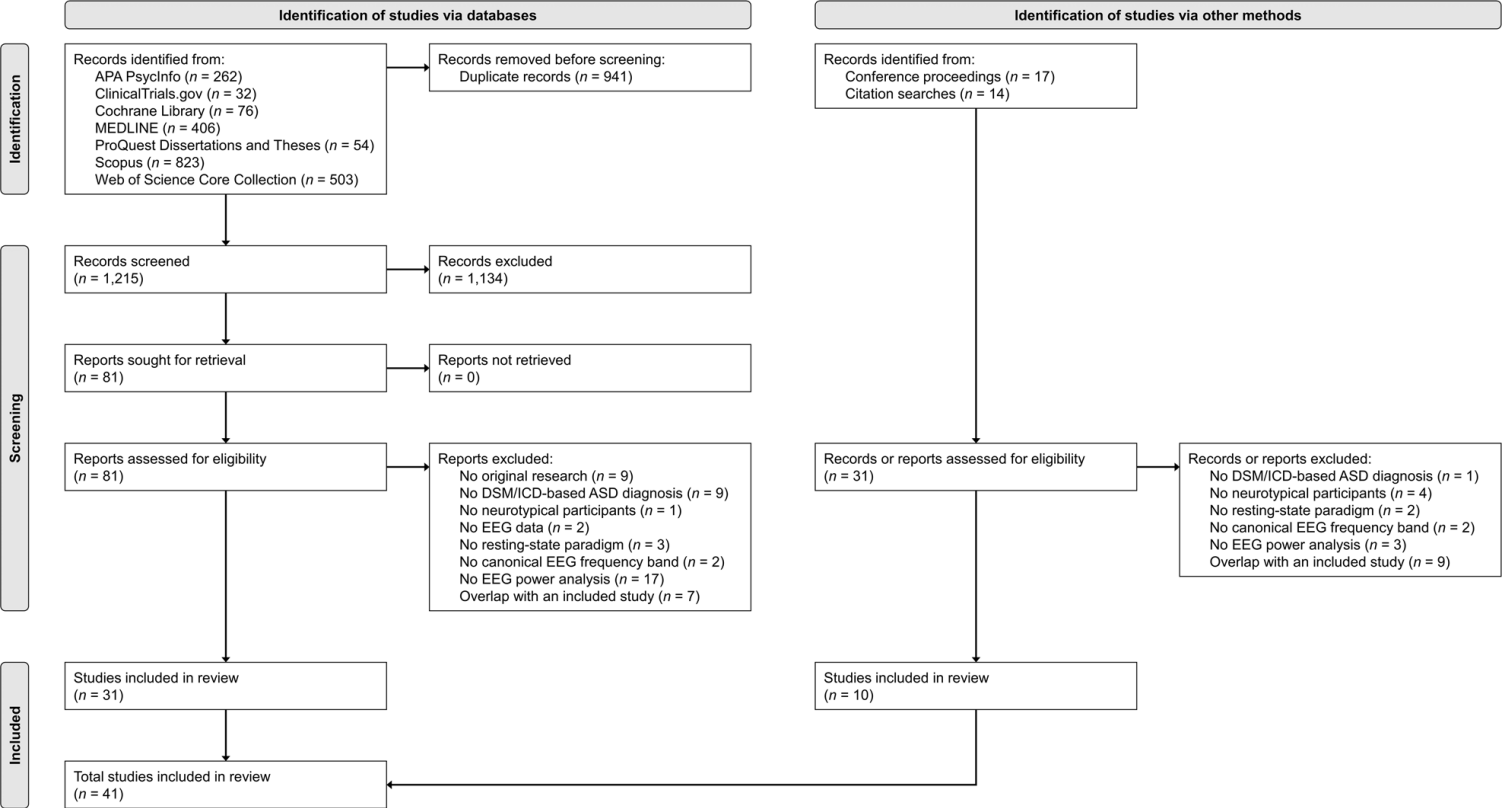
Study search and selection flow diagram. This flow diagram depicts the flow of information across the identification, screening, and inclusion phases of the systematic review.

Figure 1 depicts the results of our literature search and study selection processes. Briefly, we identified 2,187 study records from literature searches across electronic databases, conference proceedings, and citation searches. Title and abstract screening was completed for 1,215 study records. Full-text screening was completed for 112 study reports. After excluding 71 studies, this systematic review and meta-analysis included a total of 41 studies (i.e., 34 journal articles, 3 conference posters/presentations, 3 theses/dissertations, and 1 book chapter) that were published between 1986 and 2021 [37, 38, 62–100].

### Descriptive analyses

#### Sample characteristics

Key study sample characteristics are summarized in Table 1. Across studies, there were 2,701 participants (76.1% male) with a mean age of 9.9 years (range: 4.2–35.6) and mean IQ of 104.7 (range: 80.8–118.1). For the 1,246 autistic participants, 82.3% was male, mean age was 9.8 years (range: 4.3–35.5), and mean IQ was 87.0 (range: 37.5–115.7). For the 1,455 neurotypical participants, 70.7% was male, mean age was 9.8 years (range: 4.1–35.7), and mean IQ was 111.8 (range: 98.0–120.6). The percentage of studies with matched biological sex, age, and IQ was 84.2%, 97.3%, and 63.6%, respectively, suggesting that the autistic and neurotypical groups were well matched on key demographic characteristics for most studies. Clinical diagnosis of ASD was determined using DSM-5 (*k* = 9, 22.0%), DSM-IV-TR (*k* = 8, 19.5%), DSM-IV (*k* = 18, 43.9%), DSM-III-R (*k* = 2, 4.9%), DSM-III (*k* = 1, 2.4%), and ICD-10 (*k* = 10, 24.4%), reflecting the substantial period of time covered by the included studies. Fourteen (34.1%) studies used gold-standard ASD diagnostic instruments.

**Table 1:** Key study sample characteristics.

	Autistic Sample Characteristics				Neurotypical Sample Characteristics				ASD Diagnostics	
Study Authors (Year)^a^	*n*	Male, %	Age, *M* (*SD*) [Range]	IQ, *M* (*SD*) [Range]	*n*	Male, %	Age, *M* (*SD*) [Range]	IQ, *M* (*SD*) [Range]	Class System	Gold Std
Bellato et al. [62]	6	100.0	12.6 (2.2) [10.0–15.9]	107.2 (8.9) [94–116]	20	85.0	11.9 (1.8) [9.7–15.8]	111.2 (9.6) [95–127]	DSM-IV	
Bruining et al. [63]	100	73.0	10.5 (2.3) [7.0–16.0]	101.4 (20.8)	29	48.3	10.3 (1.5) [7.4–14.4]	120.6 (14.2)	DSM-5 DSM-IV-TR	ASD
Burner et al. [64]^b^	14		[6.0–7.0]		15				DSM-IV-TR	ASD
Cantor et al. [65]	11	81.8	7.9 (2.0) [4.0–12.0]	37.5 (11.4)	88		9.3 (2.5) [5.0–15.0]	113.4 (9.5) [90–129]	DSM-III	
Chan & Leung [66]	17	82.4	7.1 (2.2)	92.0 (20.9)	105	41.9	7.8 (2.0)	109.5 (15.5)	DSM-IV	
Chan et al. [67]	66	90.9	9.7 (3.0) [5.0–18.0]	83.4 (21.6)	90	53.3	8.8 (1.8) [6.0–12.0]	111.4 (16.2)	DSM-IV-TR	
Clarke et al. [68]	20	100.0	10.5 (2.1)	91.5 (14.4)	20	100.0	10.5 (2.0)	107.1 (9.9)	DSM-IV	
Coben et al. [70]	20	70.0	8.9 (2.3)	93.0 (16.8)	20	70.0	9.2 (1.2)	98.0 (15.4)	DSM-IV	
Coben et al. [69]^c^	91		9.9 (3.4)		91		[6.0–17.0]		DSM-IV	
Daoust et al. [71]	9	88.9	22.2 (4.1) [12.0–53.0]		8	87.5	23.5 (4.9) [8.0–56.0]		DSM-IV	ASD
Dawson et al. [72]	28	82.1	11.0 (4.0) [5.3–18.8]	60.0 (24.0) [20–117]	28	82.1	11.0 (4.0)		DSM-III-R	
Dickinson et al. [73]	59	78.0	5.8 (2.0) [2.1–10.5]	NV: 74.7 (33.8) [10–145] V: 69.0 (34.4) [12–160]	38	65.8	6.0 (2.2) [2.4–12.2]	NV: 112.6 (12.1) [88–156] V: 121.1 (19.4) [82–168]	DSM-IV	
Eiland et al. [74]^b^	22	81.8			37	56.8			DSM-5	ASD
Elhabashy et al. [75]	21	85.7	[4.0–12.0]		21	76.2	[4.0–12.0]		DSM-5	
Floriana [76]^d^	6	83.3	31.0 (3.0) [26.0–34.0]	77.5 (6.7) [71–89]	6	83.3	30.0 [26.0–33.0]		DSM-III-R	
Frohlich et al. [77]	10	80.0	4.3 (1.8) [2.2–8.2]	NV: 49.9 (9.2) [41–74] V: 39.6 (26.0) [17–103]	9	66.7	4.1 (0.9) [2.4–5.0]	NV: 116.9 (22.0) [94–157] V: 125.0 (16.1) [98–145]	DSM-IV	
Gabard-Durnam et al. [78]	16	71.0			49	53.6			DSM-5	ASD and NT
Gulati et al. [79]^b^	61	85.0	6.5 (1.6) [3.0–12.0]	59.9 (5.9)	48				DSM-5	
Hornung et al. [80]	32	87.5	12.7 (2.6) [7.7–17.1]	NV: 107.0 (14.0) [81–140] V: 108.0 (17.0) [72–147]	32	87.5	12.4 (2.7) [7.4–16.5]	NV: 107.0 (11.0) [88–129] V: 112.0 (11.0) [87–133]	DSM-IV	ASD
Kang et al. [81]	49	79.6	4.3 (1.1)		48	75.0	4.3 (1.0)		DSM-5	
Keehn et al. [82]	18	89.5	14.4 (1.6) [12.4–17.8]	NV: 109.0 (15.0) [81–140] V: 109.0 (19.0) [72–147]	21	81.0	14.3 (1.4) [12.0–16.8]	NV: 108.0 (10.0) [90–129] V: 107.0 (10.0) [87–126]	DSM-5	ASD
Kozhushko et al. [83]	24	87.5	5.9 (1.7) [4.0–9.0]		70	62.9	6.4 (1.4) [4.0–9.0]		ICD-10	
Lazarev et al. [84]	14	100.0	9.7 (2.3) [6.0–14.0]	91.4 (22.8)	21	100.0	10.1 (3.3) [6.0–16.0]		DSM-IV	
Lucas [85]^d^	2	100.0	[3.0–5.0]		17	63.2			DSM-IV	
Luschekina et al. [86]	27	100.0	5.8 (1.4)		24	100.0	6.1 (0.9)		ICD-10	
Lushchekina et al. [87]	31	100.0	[5.0–7.0]		25	100.0			ICD-10	
Lushchekina et al. [88]	27	100.0	5.8 (1.4)		24	100.0	6.1 (0.9)		ICD-10	
Machado et al. [89]	11	63.6	5.9 (2.4)		14	64.3	5.6 (2.5)		DSM-IV	
Mash et al. [90]	38	84.2	12.6 (2.4) [7.1–17.1]	NV: 104.0 (18.0) [64–140] V: 105.0 (18.0) [72–147]	38	76.3	13.0 (2.8) [7.1–18.0]	NV: 107.0 (12.0) [77–129] V: 108.0 (11.0) [83–126]	DSM-5	ASD and NT
Mathewson et al. [37]	15	80.0	35.5 (10.6) [18.8–51.6]	100.9 (18.6) [64–136]	16	75.0	35.7 (7.6) [22.6–47.8]	107.1 (11.9)	DSM-IV	ASD
Maxwell et al. [38]	15	100.0	15.1 (2.9) [9.0–18.0]	108.6 (16.0)	18	100.0	14.2 (2.9) [9.0–18.0]	110.7 (12.9)	DSM-IV ICD-10	ASD
Neuhaus et al. [91]	142	57.0	12.3 (2.9)	NV: 101.1 (17.7) V: 100.9 (20.6)	138	50.7	13.3 (2.9)	NV: 109.9 (15.3) V: 112.9 (16.1)	DSM-IV-TR	ASD
Orekhova et al. [92]	40	100.0	5.2 (1.4) [3.1–8.8]		40	100.0	5.3 (1.3) [3.0–7.8]		DSM-IV-TR ICD-10	
Orekhova et al. [93]	21	81.0	5.9 (1.6) [3.5–8.8]	77.4 (19.2)	20	85.7	5.9 (1.5) [4.0–8.9]		DSM-IV-TR ICD-10	
Pierce et al. [94]	31	80.6	11.3 (1.6) [6.5–14.6]	NV: 104.0 (17.0) [70–136] V: 101.0 (19.0) [67–154]	31	71.0	10.6 (1.9) [6.6–15.0]	NV: 109.0 (13.0) [87–132] V: 108.0 (12.0) [89–129]	DSM-5	ASD
Sheikhani et al. [95]	15	66.7	9.2 (2.7) [6.0–11.0]	V: 114.3 (19.9) [85–140]	11	63.6	9.1 (1.7) [6.0–11.0]	V: 111.4 (14.7) [90–135]	DSM-IV-TR	
Shephard et al. [96]	16	100.0	11.5 (1.6)	115.4 (15.5)	22	100.0	10.3 (1.9)	120.0 (13.4)	DSM-IV ICD-10	ASD
Stroganova et al. [97]	40	100.0	5.2 (1.4) [3.1–8.8]		40	100.0	5.3 (1.3) [3.0–7.8]		DSM-IV-TR ICD-10	
Sutton et al. [98]	23	82.6	11.4 (1.5)	NV: 110.1 (12.2) V: 109.7 (21.0)	20	80.0	11.3 (1.6)	NV:116.8 (11.7) V: 117.4 (15.6)	DSM-IV	
Tye [99]^d^	19	100.0	11.7 (1.7)	115.7 (15.7)	24	100.0	10.6 (1.8)	120.0 (13.4)	DSM-IV ICD-10	ASD
Van Diessen et al. [100]	19	84.2	10.6 (4.1)		19	84.2	10.1 (3.8)		DSM-IV	

*NV* nonverbal IQ, *V* verbal IQ, *ASD* autism spectrum disorder, *Class System* diagnostic classification system used for establishing, confirming, and/or ruling out clinical diagnosis of ASD, *Gold Std* use of gold-standard ASD diagnostic instruments (i.e., ADOS-2 or ADI-R or earlier editions) for autistic and/or neurotypical samples, *NT* neurotypical.

^a^Journal article unless otherwise noted by a different superscript.

^b^Conference poster/presentation.

^c^Book chapter.

^d^Thesis/dissertation.

#### EEG Parameters

Key EEG study parameters are summarized in Table 2. In terms of resting-state paradigm, 13 (31.7%) studies employed an eyes-closed paradigm and 27 (65.9%) studies employed an eyes-open paradigm. Most studies either used the mastoids (*k* = 23, 56.1%) or average (*k* = 13, 31.7%) referencing scheme. Across studies, the mean duration of individual epochs used in EEG power spectral analyses was 6.1 s (*SD* = 14.2, range: 1.0–60.0) and the mean recording duration was 5.8 minutes (*SD* = 4.6, range: 1.5–25.0). On average, the delta frequency band was defined as 1.2–3.6 Hz, theta as 3.9–7.4 Hz, alpha as 7.6–12.2 Hz, beta as 13.0–25.3 Hz, and gamma as 29.9–49.8 Hz, indicating that adjacent canonical frequency bands had minimal overlap across studies and could be considered as relatively distinct frequency bands.

**Table 2:** Key EEG study parameters.

Study Authors (Year)^a^	Paradigm	Reference	Epoch, s	Frequency Band Limits, Hz					Type and ROI of Power Spectral Analyses				
				Delta	Theta	Alpha	Beta	Gamma	Delta	Theta	Alpha	Beta	Gamma
Bellato et al. [62]	EO	Average	2.0			8.0–12.0					Abs, Rel O		
Bruining et al. [63]	EC	Average	4.0			8.0–13.0					Rel W		
Burner et al. [64]^b^		Average				8.0–10.0					Abs F		
Cantor et al. [65]	EO	Mastoids		1.5–3.5	4.0–7.5	8.0–12.0	13.0–30.0		Rel W	Rel W	Rel W	Rel W	
Chan & Leung [66]	EO	Mastoids	60.0	1.0–3.0	4.0–7.0	8.0–12.0	15.0–20.0		Abs, Rel F	Abs, Rel F	Abs, Rel F	Abs, Rel F	
Chan et al. [67]	EO	Mastoids		1.0–3.5	4.0–7.5	8.0–12.0	12.0–17.5		Abs, Rel W	Abs, Rel W	Abs, Rel W	Abs, Rel W	
Clarke et al. [68]	EC	Mastoids	1.0	1.5–3.5	3.5–7.5	7.5–12.5	12.5–25.0		Abs, Rel W	Abs, Rel W	Abs, Rel W	Abs, Rel W	
Coben et al. [70]	EC	Mastoids	2.6	1.5–3.5	3.5–7.5	7.5–12.5	12.5–25.0		Abs, Rel W	Abs, Rel W	Abs, Rel W	Abs, Rel W	
Coben et al. [69]^c^	EC	Mastoids		1.5–3.5	3.5–7.5	7.5–12.5	12.5–25.0		Abs, Rel F,C,P,T,O	Abs, Rel F,C	Rel F,C,P	Rel F,C,P	
Daoust et al. [71]	EC	Mastoids	4.0	0.8–3.5	4.0–7.8	8.0–12.8	13.0–19.8		Abs F,C,T,O	Abs F,C,T,O	Abs F,C,T,O	Abs F,C,T,O	
Dawson et al. [72]	EO	Mastoids	1.0	1.5–3.5	4.0–6.0	7.0–13.0	14.0–32.0		Abs W	Abs W	Abs W	Abs W	
Dickinson et al. [73]	EO	Average	2.0			6.0–12.0					Rel W		
Eiland et al. [74]^b^	EC					8.0–13.0					Abs O		
Elhabashy et al. [75]	EO	Mastoids	4.0	0.5–4.0	4.0–8.0	8.0–12.0	13.0–25.0		Abs, Rel F	Abs, Rel F	Abs, Rel F	Abs, Rel F	
Floriana [76]^d^	EC	Mastoids							Rel W	Rel W	Rel W	Rel W	
Frohlich et al. [77]	EO	Average	2.0				12.0–30.0					Rel W	
Gabard-Durnam et al. [78]	EO	Average	2.0	2.0–4.0	4.0–6.0	6.0–13.0	13.0–30.0	30.0–50.0	Abs F	Abs F	Abs F	Abs F	Abs F
Gulati et al. [79]^b^	EO									Abs W			
Hornung et al. [80]	EO	Average	2.0		4.0–7.5					Abs, Rel W			
Kang et al. [81]	EO	Mastoids	4.0	1.0–4.0	4.0–8.0	8.0–13.0	13.0–30.0	30.0–45.0	Rel W	Rel W	Rel W	Rel W	Rel W
Keehn et al. [82]	EO	Mastoids	1.0			8.0–12.0					Abs W		
Kozhushko et al. [83]	EO	Mastoids	4.1		4.0–8.0	8.0–13.0	13.0–30.0			Abs W	Abs W	Abs W	
Lazarev et al. [84]	EC	Mastoids	2.0	3.0–3.5	4.0–7.0	7.5–12.5	13.0–24.0		Abs O	Abs O	Abs O	Abs O	
Lucas [85]^d^	EC	Vertex	1.0			5.5–9.5			Rel F,C,T,O	Rel F,C,T,O	Rel F,C,T,O	Rel F,C,T,O	
Luschekina et al. [86]	EC	Mastoids	4.0				13.5–19.5					Abs W	
Lushchekina et al. [87]	EC	Mastoids				7.5–13.0		45.0–65.0			Abs W		Abs W
Lushchekina et al. [88]	EC	Mastoids	60.0		4.0–7.5					Abs W			
Machado et al. [89]	EO	Mastoids	5.0	1.2–3.5	3.5–7.5	7.5–11.0	15.0–25.0	25.0–55.0	Abs, Rel W	Abs, Rel W	Abs, Rel W	Abs, Rel W	Abs, Rel W
Mash et al. [90]	EO	Average				8.0–12.0					Abs, Rel O		
Mathewson et al. [37]	EO	Vertex	1.0			8.0–13.0			Abs W	Abs W	Abs W	Abs W	Abs W
Maxwell et al. [38]	EO	Nose tip	1.0					30.0–50.0					Abs W
Neuhaus et al. [91]	EO	Average	2.0	1.0–3.0	4.0–7.0	8.0–12.0	13.0–29.0	30.0–50.0	Abs F,C,P	Abs F,C,P	Abs F,C,P	Abs F,C,P	Abs F,C,P
Orekhova et al. [92]	EO	Mastoids	2.5				13.2–24.0	24.4–44.0				Abs F,C,P,T,O	Abs F,C,P,T,O
Orekhova et al. [93]	EO	Mastoids	1.0					25.0–44.0					Abs W
Pierce et al. [94]	EO	Average	1.0			8.0–12.0					Abs W		
Sheikhani et al. [95]	EO	Mastoids	3.0										Abs F,C,P,T,O
Shephard et al. [96]	EO	Average	2.0	0.5–3.5	4.0–8.0	8.0–12.0	12.0–20.0		Abs F,C,P,O	Abs F,C,P,O	Abs F,C,P,O	Abs F,C,P,O	
Stroganova et al. [97]	EO	Mastoids	2.5	1.6–3.6	4.0–7.2	7.6–12.0			Abs F,C,P,T,O	Abs W	Abs W		
Sutton et al. [98]	EO	Mastoids	1.0			8.0–13.0					Abs F,C,P		
Tye [99]^d^	EO	Average	2.0	0.5–3.5	4.0–8.0	8.0–12.0	12.0–20.0		Abs, Rel F,C,P	Abs, Rel F,C,P	Abs, Rel F,C,P	Abs, Rel F,C,P	
Van Diessen et al. [100]	EC	Average	8.0	0.5–4.0	4.0–8.0	8.0–13.0	13.0–30.0	30.0–45.0	Rel W	Rel W	Rel W	Rel W	Rel W

*EC* eyes-closed paradigm, *EO* eyes-open paradigm, *ROI* region of interest, *Abs* absolute power, *Rel* relative power, *W* whole, *F* frontal, *C* central, *P* parietal, *T* temporal, *O* occipital.

^a^Journal article unless otherwise noted by a different superscript.

^b^Conference poster/presentation.

^c^Book chapter.

^d^Thesis/dissertation.

#### Effect sizes

After ensuring independence of effect sizes, the 41 included studies yielded 147 effect sizes, of which 86 and 61 were for absolute and relative EEG power, respectively. Preliminary analyses identified 12 outlier effect sizes with absolute values of externally standardized residuals ranging from 2.12 to 6.93 (*M* = 3.57, *SD* = 1.47). These outliers were distributed across study outcomes (i.e., at most two outliers for any study outcome). Notably, three studies [66, 85, 89] contributed to the majority (75.%) of these outlier effect sizes, possibly due to substantially different sample characteristics and methodological decisions. For example, Lucas [85] included only two autistic individuals in the eyes-closed paradigm and was one of two studies that used a vertex referencing scheme; similarly, Chan and Leung [66] was the only study that used a single electrode to record resting-state EEG and was one of two studies that used an epoch duration of 60 s in EEG power spectral analyses. We excluded all 12 outlier effect sizes from subsequent analyses.^2^

### Meta-Analyses

#### Pooled Effect Sizes

**Fig. 2:**
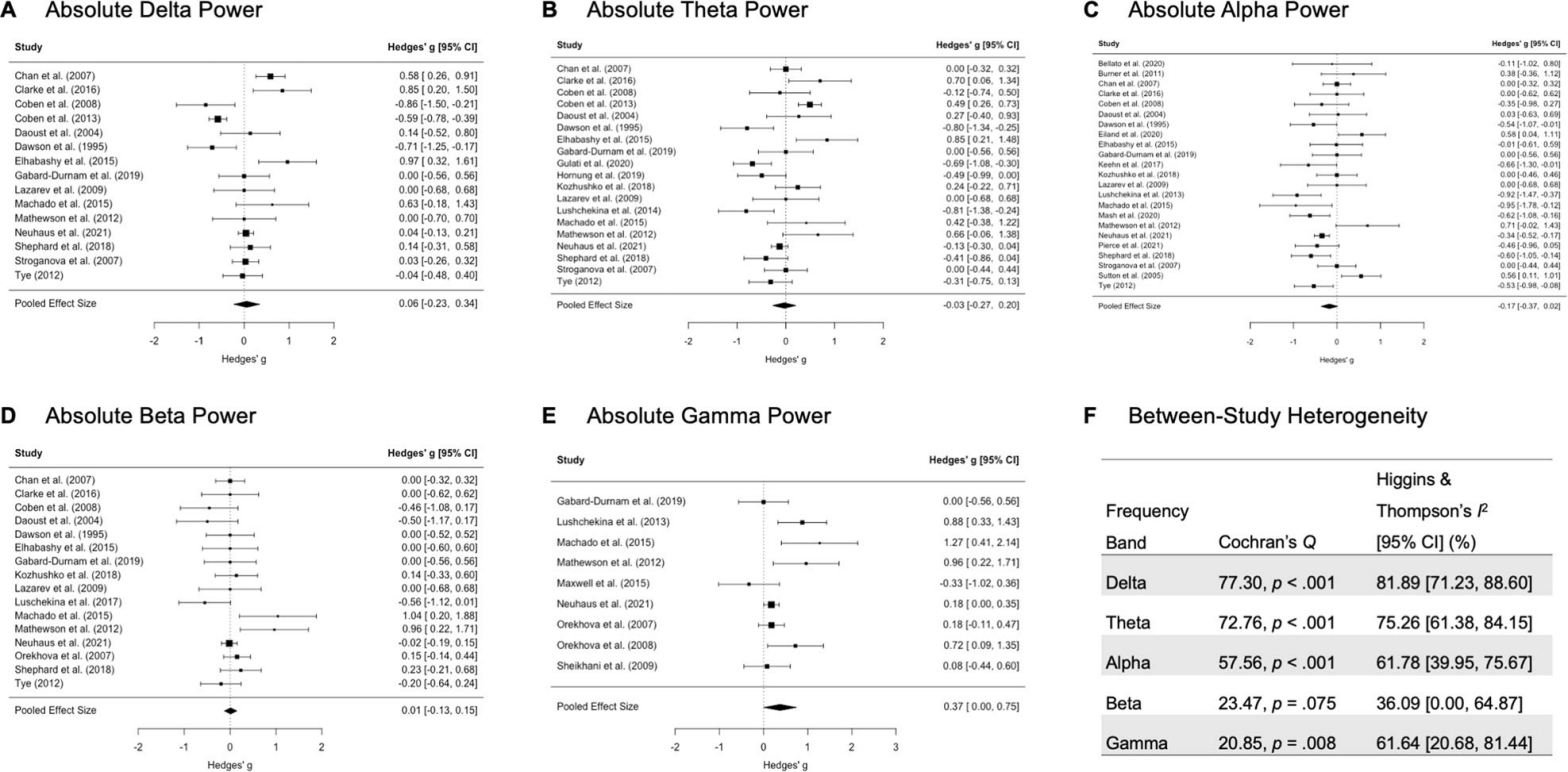
Forest plots for absolute EEG power differences. Forest plots are depicted separately for absolute (**A**) delta (*k* = 15), (**B**) theta (*k* = 19), (**C**) alpha (*k* = 23), (**D**) beta (*k* = 16), and (**E**) gamma (*k* = 9) power differences between autistic and neurotypical individuals. Positive effect sizes indicate greater absolute power in autistic individuals than neurotypical individuals. **F** Between-study heterogeneity is tabulated for all absolute power frequency bands.

**Fig. 3:**
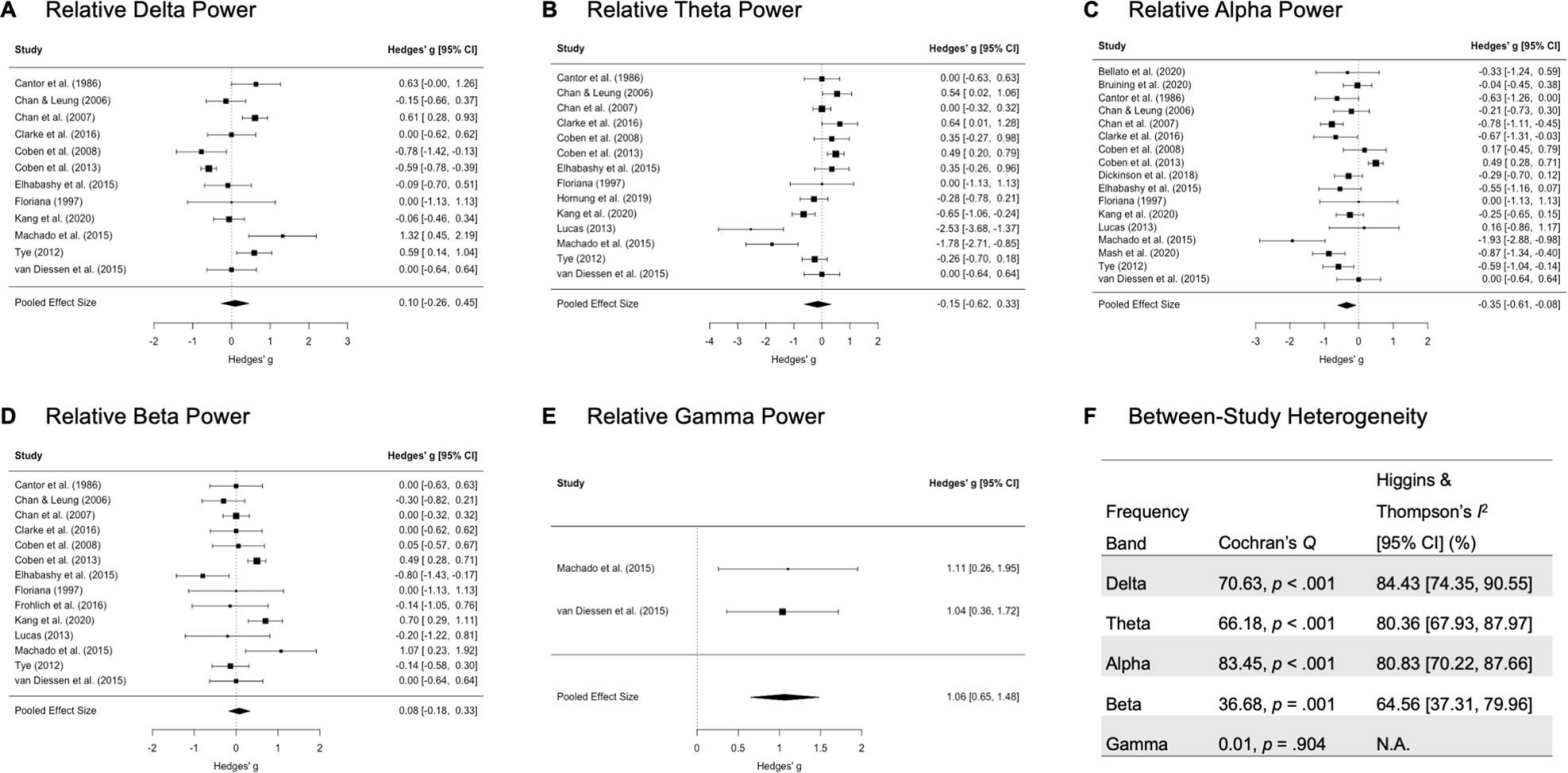
Forest plots for relative EEG power differences. Forest plots are depicted separately for relative (**A**) delta (*k* = 12), (**B**) theta (*k* = 12), (**C**) alpha (*k* = 15), (**D**) beta (*k* = 12), and (**E**) gamma (*k* = 2) power differences between autistic and neurotypical individuals. Positive effect sizes indicate greater relative power in autistic individuals than neurotypical individuals. (**F**) Between-study heterogeneity is tabulated for all relative power frequency bands.

Figures 2 and 3 show individual forest plots for absolute and relative EEG power, respectively. As expected, absolute gamma power differed between autistic and neurotypical individuals, with autistic individuals exhibiting greater power that resulted in a medium effect size, *g* = 0.37, 95% CI [0.00, 0.75], *p* = 0.049. Contrary to predictions, absolute power was similar between autistic and neurotypical individuals for the remaining frequency bands, yielding very small to small effect sizes that were nonsignificant (delta: *g* = 0.06, 95% CI [−0.23, 0.34], *p* = 0.679; theta: *g* = −0.03, 95% CI [−0.27, 0.20], *p* = 0.777; alpha: *g* = −0.17, 95% CI [−0.37, 0.02], *p* = 0.072; beta: *g* = 0.01, 95% CI [−0.13, 0.15], *p* = 0.868).

Consistent with our prediction, relative alpha power differed between autistic and neurotypical individuals, with autistic individuals exhibiting reduced power that resulted in a medium effect size, *g* = −0.35, 95% CI [−0.61, −0.08], *p* = 0.013. Additionally, as expected but with the caveat that only two studies were included in the meta-analysis, autistic individuals demonstrated greater relative gamma power than neurotypical individuals, as evidenced by a very large effect size, *g* = 1.06, 95% CI [0.65, 1.48], *p* = 0.020. Contrary to predictions, relative delta, theta, and beta power were not significantly different between autistic and neurotypical individuals, with very small to small effect sizes (delta: *g* = 0.10, 95% CI [−0.26, 0.45], *p* = 0.563; theta: *g* = −0.15, 95% CI [−0.62, 0.33], *p* = 0.515; beta: *g* = 0.08, 95% CI [−0.18, 0.33], *p* = 0.535).

#### Heterogeneity of individual study effect sizes

As tabulated in Fig. 2F, tests of heterogeneity were broadly significant across all absolute power frequency bands (*Q*s > 20.85, *p*s < 0.008), except for absolute beta power (*Q* = 23.47, *p* = 0.075). Similarly, Fig. 3F revealed significant tests of heterogeneity across all relative power frequency bands (*Q*s > 36.68, *p*s < 0.001), with the exception of relative gamma power (*Q* = 0.01, *p* = 0.904). These substantial differences across individual study effect sizes represented moderate to high levels of heterogeneity, with *I*^2^ values ranging from 36.1% to 81.9% and from 64.6% to 84.4% for absolute and relative EEG power, respectively.

### Moderator analyses

Table 3 summarizes findings of subgroup and meta-regression analyses. Broadly, these moderator analyses did not provide support for effect sizes being moderated by full-sample demographic characteristics, study methodological differences, and potential sources of bias. Specifically, full-sample biological sex, age, and IQ did not moderate effect sizes across all absolute (*F*s < 3.13, *p*s > 0.127) and relative (*F*s < 2.23, *p*s > 0.195) power frequency bands. Referencing scheme, epoch duration, and recording duration did not moderate effect sizes across all absolute (*F*s < 2.59, *p*s > 0.128) and relative (*F*s < 2.19, *p*s > 0.189) power frequency bands, with the exception that recording duration was a significant moderator of effect sizes for relative alpha power (*F* = 6.26, *p* = 0.034), such that longer recording durations were associated with negative effect sizes of larger magnitudes (*B* = − 0.06, *SE* = 0.02). Use of gold-standard ASD diagnostic instruments did not moderate all absolute power effect sizes (*F*s < 0.22, *p*s > 0.646).

**Table 3:** Moderator analyses for absolute and relative EEG power differences.

	Omnibus Test of Moderator										Meta-Regression Coefficient									
	Delta		Theta		Alpha		Beta		Gamma		Delta		Theta		Alpha		Beta		Gamma	
Moderator	*F*	*p*	*F*	*p*	*F*	*p*	*F*	*p*	*F*	*p*	*B*	*SE*	*B*	*SE*	*B*	*SE*	*B*	*SE*	*B*	*SE*
Absolute EEG Power																				
Biological sex	0.04	0.837	0.59	0.455	0.66	0.427	0.13	0.728	0.01	0.914	0.00	0.01	−0.01	0.01	−0.01	0.01	−0.00	0.00	0.00	0.01
Age	0.03	0.856	1.79	0.203	2.53	0.131	0.64	0.441	0.22	0.660	−0.00	0.02	0.02	0.02	0.02	0.02	0.01	0.02	0.01	0.02
IQ	0.00	0.977	3.13	0.127	0.38	0.552	0.09	0.778			−0.00	0.03	−0.02	0.01	−0.01	0.02	0.00	0.02		
Resting-state paradigm^a^	0.99	0.337	0.65	0.432	0.24	0.633	**4.89**	**0.044**			0.29	0.29	−0.20	0.25	−0.11	0.22	**0.38**	**0.17**		
Referencing scheme^b^			2.59	0.128	1.14	0.299							0.36	0.22	0.18	0.17				
Epoch duration	1.86	0.200	2.52	0.135	0.21	0.649	0.16	0.695	0.58	0.477	0.16	0.12	−0.01	0.01	−0.04	0.08	−0.03	0.09	0.12	0.16
Recording duration	0.56	0.472	1.96	0.187	0.92	0.353	0.44	0.522	0.00	0.968	−0.01	0.02	0.03	0.02	−0.03	0.03	−0.02	0.02	−0.01	0.29
Gold-standard ASD^c^	0.01	0.943	0.22	0.646	0.03	0.865	0.02	0.893			−0.02	0.28	−0.11	0.24	−0.03	0.19	−0.02	0.14		
Relative EEG Power																				
Biological sex	0.04	0.850	0.61	0.456	1.65	0.224	0.20	0.666			0.00	0.01	−0.01	0.01	−0.01	0.01	0.00	0.01		
Age	0.08	0.788	0.11	0.750	0.01	0.909	1.20	0.306			−0.01	0.03	0.01	0.03	−0.00	0.02	−0.02	0.02		
IQ	1.36	0.308	2.23	0.195	0.38	0.555	1.17	0.329			0.03	0.03	−0.03	0.02	−0.01	0.01	−0.01	0.01		
Resting-state paradigm^a^	**7.00**	**0.025**	4.11	0.070	**8.03**	**0.014**	0.34	0.573			**0.70**	**0.26**	−0.44	0.22	**−0.44**	**0.15**	−0.11	0.19		
Referencing scheme^b^					0.07	0.789									−0.05	0.18				
Epoch duration	0.14	0.722	1.23	0.311	0.10	0.764	1.21	0.313			−0.00	0.01	0.01	0.01	0.00	0.00	−0.01	0.01		
Recording duration	1.55	0.269	2.19	0.189	**6.26**	**0.034**	1.46	0.272			−0.03	0.02	0.03	0.02	**−0.06**	**0.02**	0.02	0.02		
Gold-standard ASD^c^																				

Significant values are shown in bold. Empty cells are due to insufficient (i.e., less than five) studies required for subgroup and meta-regression analyses with reasonable statistical power. Planned moderator analyses of matched demographic characteristics (i.e., biological sex, age, and IQ) were not conducted across all absolute and relative power frequency bands due to insufficient (i.e., less than five) studies for at least one of the two subgroups (i.e., “No” and “Yes”).

^a^Reference category: Eyes-closed resting-state paradigm.

^b^Reference category: Average referencing scheme.

^c^Reference category: No use of gold-standard ASD diagnostic instruments for neither autistic nor neurotypical groups.

Notably, moderator analyses of resting-state paradigm yielded mixed results, with significant effects for absolute beta (*F* = 4.89, *p* = 0.044), relative delta (*F* = 7.00, *p* = 0.025), and relative alpha power (*F* = 8.03, *p* = 0.014), but not for the remaining absolute and relative power frequency bands (*F*s < 4.11, *p*s > 0.070). For absolute beta power, the pooled effect sizes for studies using eyes-closed and eyes-open paradigms were *g* = −0.32, 95% CI [−0.66, 0.02], *p* = 0.067 and *g* = 0.06, 95% CI [−0.07, 0.19], *p* = 0.342, respectively. For relative delta power, the pooled effect sizes for eyes-closed and eyes-open paradigms were *g* = −0.34, 95% CI [−0.80, 0.11], *p* = 0.125 and *g* = 0.35, 95% CI [−0.01, 0.72], *p* = 0.058, respectively. For relative alpha power, the pooled effect sizes for eyes-closed and eyes-open paradigms were *g* = −0.09, 95% CI [−0.36, 0.19], *p* = 0.523 and *g* = −0.52, 95% CI [−0.70, −0.34], *p* < 0.001, respectively.

## Discussion

We conducted a systematic review and meta-analysis of 41 resting-state EEG studies that examined absolute and relative power differences in ASD across five canonical delta, theta, alpha, beta, and gamma bands, based on a total sample of more than 2,700 autistic and neurotypical individuals and 135 effect sizes. To our knowledge, the present study is the first to (a) quantitatively synthesize the contemporary literature on resting-state EEG power differences in ASD using meta-analytic approaches; (b) conscientiously include a substantial amount of gray literature in accordance with PRISMA guidelines; and (c) systematically evaluate several potential moderators that may account for heterogeneous study findings.

We observed three major findings. First, as expected, our meta-analyses demonstrated that autistic individuals exhibit greater absolute and relative resting-state gamma power than neurotypical individuals with medium to very large effect sizes. Consistent with our predictions, relative resting-state alpha power is reduced in ASD, yielding a medium effect size. In contrast, we found limited evidence of EEG power differences between autistic and neurotypical individuals for delta, theta, beta, and absolute alpha power. Broadly, our meta-analyses across all five canonical EEG frequency bands offered some support for the U-shaped profile of resting-state EEG power differences described in prior narrative reviews [36]. Additionally, the significant findings for gamma and alpha bands are largely compatible with theoretical accounts that disruptions to GABA neurotransmitter systems are implicated in the pathogenesis of ASD and reflected in neural networks being biased toward gamma-related excitation and away from alpha-related inhibition [36, 101, 102]. Nevertheless, current findings for relative gamma power were based on only two studies, thus warranting replication efforts. Additionally, gamma oscillations overlap considerably with muscle activity, thus resting-state gamma power is susceptible to various muscle artifacts (e.g., microsaccades). Therefore, specific techniques to optimize acquisition and analysis of resting-state gamma power as well as to detect and remove muscle artifacts during preprocessing may be needed when leveraging gamma power metrics for clinical purposes [103, 104].

Second, we obtained significantly heterogeneous study effect sizes across most absolute and relative power frequency bands. Despite moderate to high levels of heterogeneity, moderator analyses of key demographic characteristics, methodological details, and risk-of-bias indicators largely resulted in null findings. A likely explanation is that our moderator analyses might have been statistically underpowered, as is relatively common in moderator analyses of meta-analytic models [105]. An alternative perspective is that resting-state EEG power differences in ASD may be robust to variability in demographic characteristics. Supporting this potential interpretation, past studies were quite variable in their sampling approaches. For example, several within-study samples included participants with wide IQ (e.g., 64 to 136 for the autistic sample in [37]) and age ranges (e.g., 8 to 56 years for the neurotypical sample in [71]); samples across studies differed considerably in biological sex (e.g., approximately balanced male and female sample in [91] and exclusively male sample in [68]) and age (e.g., young children in [77] and adults in [37]). If these null findings are replicated, it is possible that resting-state gamma and relative alpha power differences may hold true even for infants and toddlers of both sexes, holding promise for potential applications of resting-state EEG power as an ASD biomarker that could be applied to support diagnosis at early ages. Nevertheless, it is important to recognize that EEG frequency bands in infancy differ from those in late childhood and adulthood [23, 106]. Further work may clarify how best to operationalize gamma and alpha frequency bands across a broad range of ages.

Finally, we observed that resting-state paradigm type and recording duration significantly moderated some results. Specifically, for absolute beta and relative delta power, eyes-open paradigms yielded positive effect sizes, while eyes-closed paradigms resulted in negative effect sizes. For relative alpha power, eyes-open paradigms and longer recording durations resulted in negative effect sizes of larger magnitudes. These patterns of results suggest that predicted differences in resting-state EEG power in ASD may be optimally detected in eyes-open and sufficiently long paradigms, raising important translational questions on how best to deploy such paradigms in clinical contexts while balancing practical considerations (e.g., increased likelihood of participant noncompliance with prolonged protocols).

Although the present study has made several methodological advancements relative to prior narrative reviews, our findings must be considered in the context of the relatively small number of studies that were available in the literature to be synthesized. The small number of included studies likely resulted in suboptimal subgroup and meta-regression moderator analyses. One potential solution to amass an expanded set of resting-state neurophysiological studies involving autistic and neurotypical individuals is to integrate magnetoencephalography (MEG) studies with comparable resting-state paradigms, though special considerations are needed when interpreting MEG findings due to fixed sensor locations (e.g., MEG-specific motion artifacts) and when synthesizing across EEG and MEG studies [107]. We were also unable to examine a number of EEG study parameters that are relevant to power spectral analyses but have not been consistently explored in previous studies. For example, during the transformation of EEG signals from the time to frequency domain, characteristics of taper window functions directly impact the computation of resting-state EEG power but are often reported with incomplete details [108]. Increased adoption of recommended reporting standards for electrophysiological research in ASD may pave the way for more robust investigations into potential moderators in future studies [107].

In summary, the present systematic review and meta-analysis provides an updated and, for the first time, quantitative synthesis of published and gray literature on resting-state EEG power differences in ASD. We found that autistic individuals exhibit reduced relative alpha power and increased gamma power, offering initial evidence that resting-state alpha and gamma power metrics may be promising ASD biomarker candidates. Future studies may consider examining additional diagnostic and psychometric properties of resting-state alpha and gamma power, such as reliability, sensitivity, and specificity. It will also be important to further evaluate whether resting-state alpha and gamma power differences reported in the present study are unique to ASD, given resting-state EEG power differences in neurodevelopmental and neurogenetic disorders that frequently cooccur with ASD, such as attention-deficit/hyperactivity disorder, Down syndrome, and fragile X syndrome [39, 109, 110]. Since the present study focused on autistic and neurotypical individuals, a natural extension is to synthesize the emerging literature on resting-state EEG power differences between young children at low and high likelihood of receiving an ASD diagnosis. Additionally, to fully realize the efficacy of resting-state EEG power for clinical diagnostics, it will be valuable to optimize neurodiagnostic clinical protocols, including streamlining resting-state EEG acquisition procedures, automating components of preprocessing and analytic pipelines, and integrating EEG power metrics into broader diagnostic frameworks. Broadly, the present study holds clinical translational value and may contribute to ongoing efforts aimed at incorporating psychophysiological markers into multi-tiered assessment approaches for early diagnosis of ASD.

### Supplementary information

Supplementary Materials

## Footnotes

1 Given that EEG power differences may be specific to topographical regions, we conducted supplemental analyses that did not aggregate effect sizes across regions of interest (Supplementary Table 2). To ensure robustness, we also conducted two sensitivity analyses that assumed small-sized and large-sized correlations of .1 and .5, respectively, in the compound symmetric variance-covariance matrix (Supplementary Table 3).

2 Given that we did not include identification of outlier effect sizes in our preregistration, we conducted parallel meta-analyses without excluding any effect sizes and obtained qualitatively similar results.
