# Supplementary material for: Resting-state EEG power differences in autism spectrum disorder: a systematic review and meta-analysis
Source: Transl Psychiatry. 2023 Dec 14;13:389. doi: 10.1038/s41398-023-02681-2 (PMC10721649; doi:10.1038/s41398-023-02681-2)
Supplement: Supplementary file 1 — Supplementary Materials [file 41398_2023_2681_MOESM1_ESM.docx]

**Supplementary Materials**

**Line-by-Line Search Strategies**

We conducted comprehensive literature searches in several information sources. Line-by-line search strategies for each individual information source are detailed below.

***APA PsycInfo (EBSCO)***

1. TI (ASD OR PDD OR autis* OR “pervasive development*” OR Asperger*)
2. AB (ASD OR PDD OR autis* OR “pervasive development*” OR Asperger*)
3. KW (ASD OR PDD OR autis* OR “pervasive development*” OR Asperger*)
4. DE “Autism Spectrum Disorders”
5. 1 OR 2 OR 3 OR 4
6. TI ((rest* OR baseline OR oscillat* OR quantitative OR spontaneous) AND (EEG OR qEEG OR electroencephal* OR electrophysio* OR (DE “Electroencephalography”) OR (DE “Neurotherapy”)))
7. AB ((rest* OR baseline OR oscillat* OR quantitative OR spontaneous) AND (EEG OR qEEG OR electroencephal* OR electrophysio* OR (DE “Electroencephalography”) OR (DE “Neurotherapy”)))
8. KW ((rest* OR baseline OR oscillat* OR quantitative OR spontaneous) AND (EEG OR qEEG OR electroencephal* OR electrophysio* OR (DE “Electroencephalography”) OR (DE “Neurotherapy”)))
9. 6 OR 7 OR 8
10. 5 AND 9

Filters of “English” for language and “Human” for population group were applied.

***Cochrane Library***

1. (ASD OR PDD OR autis* OR “pervasive development*” OR Asperger*):ti,ab,kw
2. MeSH descriptor: [Child Development Disorders, Pervasive] explode all trees
3. 1 OR 2
4. (rest* OR baseline OR oscillat* OR quantitative OR spontaneous):ti,ab,kw
5. MeSH descriptor: [Rest] explode all trees
6. 4 OR 5
7. (EEG OR qEEG OR electroencephal* OR electrophysio*):ti,ab,kw
8. MeSH descriptor: [Electroencephalography] explode all trees
9. MeSH descriptor: [Neurofeedback] explode all trees
10. 7 OR 8 OR 9
11. 6 AND 10
12. 3 AND 11

***MEDLINE (PubMed)***

1. “ASD”[Title/Abstract] OR “PDD”[Title/Abstract] OR “autis*”[Title/Abstract] OR “pervasive development*”[Title/Abstract] OR “Asperger*”[Title/Abstract] OR “child development disorders, pervasive”[MeSH Terms]
2. (“rest*”[Title/Abstract] OR “baseline”[Title/Abstract] OR “oscillat*”[Title/Abstract] OR “quantitative”[Title/Abstract] OR “spontaneous”[Title/Abstract] OR “Rest”[MeSH Terms]) AND (“EEG”[Title/Abstract] OR “qEEG”[Title/Abstract] OR “electroencephal*”[Title/Abstract] OR “electrophysio*”[Title/Abstract] OR “Electroencephalography”[MeSH Terms] OR “Neurofeedback”[MeSH Terms])
3. 1 AND 2

Filters of “English” for language and “Humans” for species were applied.

***Scopus***

1. TITLE-ABS-KEY(ASD OR PDD OR autis* OR “pervasive development*” OR Asperger*)
2. TITLE-ABS-KEY(rest* OR baseline OR oscillat* OR quantitative OR spontaneous) AND TITLE-ABS-KEY(EEG OR qEEG OR electroencephal* OR electrophysio*)
3. 1 AND 2

Filters of “English” for language and INDEXTERMS(human*) were applied.

***Web of Science Core Collection***

1. TI=(ASD OR PDD OR autis* OR “pervasive development*” OR Asperger*)
2. AB=(ASD OR PDD OR autis* OR “pervasive development*” OR Asperger*)
3. AK=(ASD OR PDD OR autis* OR “pervasive development*” OR Asperger*)
4. 1 OR 2 OR 3
5. TI=((rest* OR baseline OR oscillat* OR quantitative OR spontaneous) AND (EEG OR qEEG OR electroencephal* OR electrophysio*))
6. AB=((rest* OR baseline OR oscillat* OR quantitative OR spontaneous) AND (EEG OR qEEG OR electroencephal* OR electrophysio*))
7. AK=((rest* OR baseline OR oscillat* OR quantitative OR spontaneous) AND (EEG OR qEEG OR electroencephal* OR electrophysio*))
8. 5 OR 6 OR 7
9. 4 AND 8

A filter of “English” for language was applied.

***ClinicalTrials.gov***

We conducted literature searches using the search interface at <https://clinicaltrials.gov>. For the “Condition or disease” search box, we entered: ASD OR PDD OR autis* OR “pervasive development*” OR Asperger*. For the “Other terms” search box, we entered: (rest* OR baseline OR oscillat* OR quantitative OR spontaneous) AND (EEG OR qEEG OR electroencephal* OR electrophysio*).

***ProQuest Dissertations and Theses***

1. TI,AB,IF(ASD OR PDD OR autis* OR “pervasive development*” OR Asperger*)
2. TI,AB,IF((rest* OR baseline OR oscillat* OR quantitative OR spontaneous) AND (EEG OR qEEG OR electroencephal* OR electrophysio*))
3. 1 AND 2

A filter of “English” for language was applied.

***Conference Proceedings***

For annual conferences organized by the International Society for Autism Research, we downloaded electronically available conference proceedings from 2004 to 2021. We searched for the following terms individually to identify EEG studies: EEG, electroencephal, and electrophysio.

For annual conferences organized by the Society for Neuroscience, we used search interfaces associated with individual annual conferences from 2006 to 2021. For annual conferences organized by the Society for Psychophysiological Research, we downloaded electronically available conference proceedings from 2001 to 2021. We searched for the following terms individually to identify ASD studies: ASD, PDD, autis, pervasive development, and Asperger.

**Individual Data Items**

We extracted individual data items related to study metadata, sample characteristics, EEG recording, preprocessing, and spectral analysis parameters, and EEG power metrics. Definitions for individual data items are provided below.

***Study Metadata***

Definitions for study metadata are based on APA Style, which is detailed in the seventh edition of the *Publication Manual of the American Psychological Association*.

- Study author(s): Per definition in Section 9.7 and in parenthetical citation format
- Publication year: Per definition in Section 9.13 and in year only format
- Study title: Per definition in Section 9.18
- Publication source: Per definition in Section 9.23
- Publication type: One of the following options – book chapter; conference poster/presentation; journal article; thesis/dissertation; or others

***Sample Characteristics***

Each of the following sample characteristics was coded separately for the autistic and neurotypical groups.

- Sample size: Number of individuals that EEG power metrics were based on
- Sample size (demographic data): Number of individuals that demographic data were based on
- Biological sex: Percentage of individuals who were male
- Age (*M*): Mean age in years
- Age (*SD*): Standard deviation of age in years
- Age (minimum): Minimum age in years
- Age (maximum): Maximum age in years
- Overall IQ (*M*): Mean overall IQ
- Overall IQ (*SD*): Standard deviation of overall IQ
- Overall IQ (minimum): Minimum overall IQ
- Overall IQ (maximum): Maximum overall IQ
- Nonverbal IQ (*M*): Mean nonverbal IQ
- Nonverbal IQ (*SD*): Standard deviation of nonverbal IQ
- Nonverbal IQ (minimum): Minimum nonverbal IQ
- Nonverbal IQ (maximum): Maximum nonverbal IQ
- Verbal IQ (*M*): Mean verbal IQ
- Verbal IQ (*SD*): Standard deviation of verbal IQ
- Verbal IQ (minimum): Minimum verbal IQ
- Verbal IQ (maximum): Maximum verbal IQ
- IQ measure(s): IQ measure(s) used for assessing overall, nonverbal, and/or verbal IQ
- ASD diagnostic classification system(s): Diagnostic classification system(s) used for establishing and/or ruling out clinical diagnosis of ASD, with following possible options – DSM-5; DSM-IV-TR; DSM-IV; DSM-III-R; DSM-III; ICD-11; ICD-10
- Use of ADOS: “Yes” if any edition of the Autism Diagnostic Observation Schedule was used for establishing, confirming, and/or ruling out ASD; “No” otherwise
- Use of ADI: “Yes” if any edition of the Autism Diagnostic Interview was used for establishing, confirming, and/or ruling out ASD; “No” otherwise

Some studies might have explicitly matched the autistic and neurotypical groups on biological sex, age, and/or IQ. Other studies might have assessed whether the autistic and neurotypical groups were statistically equivalent on these demographic variables, based on *p*-values.

- Matched biological sex: “Yes” if autistic and neurotypical groups were reported and/or assessed to be matched on biological sex or if sufficient statistical information was available to determine statistical equivalence based on *p*-values; “No” otherwise
- Matched age: “Yes” if autistic and neurotypical groups were reported and/or assessed to be matched on age or if sufficient statistical information was available to determine statistical equivalence based on *p*-values; “No” otherwise
- Matched IQ: “Yes” if autistic and neurotypical groups were reported and/or assessed to be matched on overall, nonverbal, or verbal IQ or if sufficient statistical information was available to determine statistical equivalence based on *p*-values; “No” otherwise

***EEG Recording, Preprocessing, and Spectral Analysis Parameters***

- Resting-state paradigm: One of the following options – eyes-closed; eyes-open
- Number of electrodes: Number of scalp electrodes used to record resting-state EEG
- Sampling rate: Frequency used to record resting-state EEG in Hz
- Use of high-pass filter: “Yes” if online and/or offline high-pass filter was used; “No” otherwise
- High-pass filter frequency: Frequency of high-pass filter in Hz
- Use of low-pass filter: “Yes” if online and/or offline low-pass filter was used; “No” otherwise
- Low-pass filter frequency: Frequency of low-pass filter in Hz
- Use of notch filter: “Yes” if online and/or offline notch filter was used; “No” otherwise
- Notch filter frequency: Frequency of notch filter in Hz
- Referencing scheme: Re-referencing electrode/scheme if offline referencing was used or online reference electrode/scheme if offline referencing was not used
- Epoch duration: Duration of individual epochs used in spectral analyses in seconds

***EEG Power Metrics***

- Power type: One of the following options – absolute; relative
- Canonical frequency band: One of the following options – delta; theta; alpha; beta; gamma
- Region of interest: One of the following options – global; frontal; central; parietal; temporal; occipital

Each of the following EEG power data was coded separately for each combination of power type, canonical frequency band, and region of interest.

- Lower limit of frequency band: Frequency used to define lower limit of canonical frequency band in Hz
- Upper limit of frequency band: Frequency used to define upper limit of canonical frequency band in Hz
- Raw value of power metric for autistic group (*M*): Mean absolute or relative power for autistic group
- Raw value of power metric for autistic group (*SD*): Standard deviation of absolute or relative power for autistic group
- Raw value of power metric for neurotypical group (*M*): Mean absolute or relative power for neurotypical group
- Raw value of power metric for neurotypical group (*SD*): Standard deviation of absolute or relative power for neurotypical group
- Effect size of power differences between autistic and neurotypical groups: Cohen’s *d* for absolute or relative power differences between autistic and neurotypical groups; “0” if absolute or relative power was not statistically different between autistic and neurotypical groups
- *t*-value of power differences between autistic and neurotypical groups: Value of test statistic associated with *t*-test on absolute or relative power differences between autistic and neurotypical groups
- *F*-value of power differences between autistic and neurotypical groups: Value of test statistic associated with *F*-test on absolute or relative power differences between autistic and neurotypical groups
- *p*-value of power differences between autistic and neurotypical groups: *p*-value associated with inferential test on absolute or relative power differences between autistic and neurotypical groups
- Sign of power differences between autistic and neurotypical groups: “1” if absolute or relative power was greater in autistic group; “-1” if absolute or relative power was smaller in autistic group; only applicable for studies reporting *F*-value or *p*-value

**Funnel Plot Asymmetry Analyses**

We used contour-enhanced funnel plots to visually assess for asymmetrical patterns and publication bias; a contour-enhanced funnel plot depicts individual studies as a function of both magnitude and standard error of effect sizes, with shaded regions overlaid to indicate several common statistical significance levels. To statistically evaluate funnel plot asymmetry, we used Egger’s regression tests, with *p*-values less than .05 indicative of asymmetry. For study syntheses with significant asymmetry, we used Duval and Tweedie’s trim and fill method to recompute bias-adjusted pooled effect sizes.

Individual contour-enhanced funnel plots for absolute and relative power frequency bands are presented in Supplementary Figures 1 and 2, respectively. Across all absolute power frequency bands, no obvious asymmetrical patterns were visually observed, as evidenced by largely balanced distributions of individual studies around pooled effect sizes. Egger’s regression tests confirmed the absence of funnel plot asymmetry (delta: *t*(13) = 0.87, *p* = .399; theta: *t*(17) = 0.94, *p* = .360; alpha: *t*(21) = 0.41, *p* = .688; beta: *t*(14) = 0.38, *p* = .713; gamma: *t*(7) = 1.35, *p* = .218). Visual inspection suggested no obvious asymmetrical patterns across most relative power frequency bands, which were substantiated by non-statistically significant Egger’s regression tests (delta: *t*(10) = 0.85, *p* = .416; alpha: *t*(15) = −0.95, *p* = .358; beta: *t*(12) = −0.77, *p* = .455). In contrast, for relative theta power, the greater number of studies to the right of the pooled effect size plausibly indicated funnel plot asymmetry. Indeed, the corresponding Egger’s regression test was statistically significant, *t*(12) = −2.22, *p* = .047. The trim and fill method further estimated that three studies were missing to the left of the pooled effect size for relative theta power, which might reflect some publication bias. Imputing these missing studies resulted in a bias-adjusted pooled effect size of *g* = −0.37, 95% CI [−0.76, 0.03], *p* = .072.

**Supplementary Table 1**

*Summary of Existing Reviews on Studies of Resting-State EEG Power in Autism*

| Authors | Billeci, Sicca, Maharatna, Apicella, Narzisi, Campatelli, Calderoni, Pioggia, & Muratori | Wang, Barstein, Ethridge, Mosconi, Takarae, & Sweeney | Gurau, Bosl, & Newton | Newson & Thiagarajan | McVoy, Lytle, Fulchiero, Aebi, Adeleye, & Sajatovic |
| --- | --- | --- | --- | --- | --- |
| Publication date | August 2013 | September 2013 | July 2017 | January 2019 | September 2019 |
| Title | On the application of quantitative EEG for characterizing autistic brain: A systematic review | Resting state EEG abnormalities in autism spectrum disorders | How useful is electroencephalography in the diagnosis of autism spectrum disorders and the delineation of subtypes: A systematic review | EEG frequency bands in psychiatric disorders: A review of resting state studies | A systematic review of quantitative EEG as a possible biomarker in child psychiatric disorders |
| Search sources | Unreported | Unreported | Embase, PsycInfo, PubMed  (1980 to May 2016) | PubMed  (1993 to May 2018) | CINAHL, Cochrane, PubMed  (1996 to 2017) |
| Relevant search terms | Unreported | Unreported | ASD, Asperger, autism, EEG, encephalography, spectral analysis | Quantitative, qEEG, ongoing, on-going, spontaneous, resting, rest, EEG, ASD, autism | Quantitative EEG, ASD |
| Synthesis methods | Narrative synthesis: Yes  Meta-analysis: No | Narrative synthesis: Yes  Meta-analysis: No | Narrative synthesis: Yes  Meta-analysis: No | Vote counting: Yes  Meta-analysis: No | Narrative synthesis: Yes  Meta-analysis: No |
| Included resting-state studies with absolute and/or relative EEG power metrics | Cantor et al. (1986)  Dawson et al. (1995)  Sutton et al. (2005)  Chan et al. (2007)  Murias et al. (2007)  Orekhova et al. (2007)  Stroganova et al. (2007)  Coben et al. (2008)  Sheikhani et al. (2009)  Pop-Jordanova et al. (2010)  Lushchekina et al. (2012)  Mathewson et al. (2012) | Cantor et al. (1986)  Dawson et al. (1995)  Daoust et al. (2004)  Sutton et al. (2005)  Chan et al. (2007)  Murias et al. (2007)  Orekhova et al. (2007)  Stroganova et al. (2007)  Coben et al. (2008)  Lazarev et al. (2009)  Pop-Jordanova et al. (2010) | Cantor et al. (1986)  Dawson et al. (1995)  Daoust et al. (2004)  Chan & Leung (2006)  Chan et al. (2007)  Stroganova et al. (2007)  Sheikhani et al. (2009)  Lushchekina et al. (2012)  Mathewson et al. (2012)  Lushchekina et al. (2014)  Elhabashy et al. (2015)  Machado et al. (2015)  Matlis et al. (2015)  Maxwell et al. (2015)  van Diessen et al. (2015) | Dawson et al. (1995)  Sutton et al. (2005)  Chan & Leung (2006)  Chan et al. (2007)  Orekhova et al. (2007)  Stroganova et al. (2007)  Coben et al. (2008)  Burnette et al. (2011)  Mathewson et al. (2012)  Machado et al. (2015)  Maxwell et al. (2015)  van Diessen et al. (2015)  Kozhushko et al. (2018)  Lefebvre et al. (2018) | Chan & Leung (2006)  Coben et al. (2008)  Chan et al. (2009)  Machado et al. (2015) |

**Supplementary Table 2**

*Meta-Analytic Models by Topographical Region*

|  |  | Pooled Effect Size | | | Between-Study Heterogeneity | | | |
| --- | --- | --- | --- | --- | --- | --- | --- | --- |
| Region | *k* | Hedges’ *g* [95% CI] | *t* | *p* | τ^2^ (*SE*) | Cochran’s *Q* | *p* | *I*^2^ (%) |
| Absolute Delta Power | | | | | | | | |
| Whole | 15 | 0.06 [−0.23, 0.34] | 0.42 | .679 | 0.19 (0.10) | 77.30 | < .001 | 81.9 |
| Frontal | 8 | 0.09 [−0.29, 0.46] | 0.55 | .601 | 0.14 (0.11) | 26.22 | .001 | 73.3 |
| Central | 6 | −0.04 [−0.42, 0.35] | −0.24 | .820 | 0.09 (0.10) | 15.38 | .009 | 67.5 |
| Parietal | 5 | −0.22 [−0.56, 0.13] | −1.74 | .157 | 0.05 (0.07) | 10.64 | .031 | 62.4 |
| Temporal | 3 | −0.23 [−1.24, 0.78] | −0.97 | .436 | 0.12 (0.19) | 6.19 | .045 | 67.7 |
| Occipital | 5 | −0.17 [−0.58, 0.24] | −1.14 | .318 | 0.07 (0.11) | 8.40 | .078 | 52.4 |
| Absolute Theta Power | | | | | | | | |
| Whole | 19 | −0.03 [−0.27, 0.20] | −0.29 | .777 | 0.16 (0.08) | 72.76 | < .001 | 75.3 |
| Frontal | 7 | 0.13 [−0.28, 0.55] | 0.78 | .465 | 0.12 (0.11) | 19.55 | .003 | 69.3 |
| Central | 5 | −0.00 [−0.48, 0.48] | −0.01 | .990 | 0.11 (0.12) | 15.20 | .004 | 73.7 |
| Parietal | 3 | −0.28 [−0.89, 0.34] | −1.93 | .194 | 0.03 (0.09) | 2.54 | .281 | 21.3 |
| Temporal^a^ | 1 |  |  |  |  |  |  |  |
| Occipital | 3 | −0.07 [−0.31, 0.18] | −1.14 | .372 | 0.00 (0.14) | 0.15 | .930 |  |
| Absolute Alpha Power | | | | | | | | |
| Whole | 23 | *−0.17 [−0.37, 0.02]* | *−1.89* | *.072* | 0.11 (0.06) | 57.56 | < .001 | 61.8 |
| Frontal | 8 | −0.12 [−0.43, 0.18] | −0.97 | .364 | 0.05 (0.07) | 11.30 | .126 | 38.1 |
| Central | 5 | −0.18 [−0.79, 0.43] | −0.83 | .455 | 0.14 (0.17) | 10.38 | .035 | 61.5 |
| Parietal | 4 | −0.21 [−1.17, 0.76] | −0.68 | .546 | 0.27 (0.28) | 11.80 | .008 | 74.6 |
| Temporal^a^ | 1 |  |  |  |  |  |  |  |
| Occipital | 6 | −0.17 [−0.69, 0.34] | −0.86 | .429 | 0.16 (0.18) | 13.37 | .020 | 62.6 |
| Absolute Beta Power | | | | | | | | |
| Whole | 16 | 0.01 [−0.13, 0.15] | 0.17 | .868 | 0.00 (0.01) | 23.47 | .075 | 36.1 |
| Frontal | 7 | 0.01 [−0.13, 0.15] | 0.18 | .863 | 0.00 (0.03) | 2.63 | .854 |  |
| Central | 5 | 0.04 [−0.34, 0.41] | 0.26 | .806 | 0.02 (0.05) | 5.81 | .214 | 31.1 |
| Parietal | 4 | −0.01 [−0.20, 0.19] | −0.10 | .923 | 0.00 (0.04) | 1.23 | .745 |  |
| Temporal | 2 | −0.04 [−1.29, 1.20] | −0.46 | .727 | 0.00 (0.20) | 0.23 | .631 |  |
| Occipital | 4 | 0.09 [−0.39, 0.57] | 0.57 | .606 | 0.00 (0.08) | 2.82 | .420 |  |
| Absolute Gamma Power | | | | | | | | |
| Whole | 9 | **0.37 [0.00, 0.75]** | **2.32** | **.049** | 0.12 (0.10) | 20.85 | .008 | 61.6 |
| Frontal | 4 | 0.12 [−0.06, 0.31] | 2.12 | .124 | 0.00 (0.04) | 1.07 | .784 |  |
| Central | 3 | 0.29 [−0.33, 0.92] | 2.01 | .183 | 0.02 (0.07) | 2.86 | .240 | 30.0 |
| Parietal | 3 | **0.20 [0.02, 0.38]** | **4.76** | **.041** | 0.00 (0.04) | 0.34 | .843 |  |
| Temporal^b^ | 2 |  |  |  |  |  |  |  |
| Occipital^b^ | 2 |  |  |  |  |  |  |  |
| Relative Delta Power | | | | | | | | |
| Whole | 12 | 0.10 [−0.26, 0.45] | 0.60 | .563 | 0.23 (0.13) | 70.63 | < .001 | 84.4 |
| Frontal | 4 | −0.13 [−0.85, 0.58] | −0.60 | .590 | 0.14 (0.17) | 10.84 | .013 | 72.3 |
| Central | 2 | 0.05 [−8.39, 8.49] | 0.07 | .954 | 0.82 (1.25) | 14.31 | < .001 | 93.0 |
| Parietal | 2 | −0.06 [−7.18, 7.06] | −0.11 | .933 | 0.57 (0.89) | 10.45 | .001 | 90.4 |
| Temporal^a^ | 1 |  |  |  |  |  |  |  |
| Occipital^a^ | 1 |  |  |  |  |  |  |  |
| Relative Theta Power | | | | | | | | |
| Whole | 14 | −0.15 [−0.62, 0.33] | −0.67 | .515 | 0.45 (0.22) | 66.18 | < .001 | 80.4 |
| Frontal | 5 | −0.09 [−1.54, 1.35] | −0.18 | .869 | 0.83 (0.69) | 18.19 | .001 | 78.0 |
| Central | 2 | −1.30 [−16.30, 13.71] | −1.10 | .470 | 2.41 (4.00) | 6.77 | .009 | 85.2 |
| Parietal^a^ | 1 |  |  |  |  |  |  |  |
| Temporal^a^ | 1 |  |  |  |  |  |  |  |
| Occipital^a^ | 1 |  |  |  |  |  |  |  |
| Relative Alpha Power | | | | | | | | |
| Whole | 17 | **−0.35 [−0.61, −0.08]** | **−2.80** | **.013** | 0.17 (0.09) | 83.45 | < .001 | 80.8 |
| Frontal | 5 | −0.11 [−0.69, 0.46] | −0.55 | .610 | 0.17 (0.19) | 15.78 | .003 | 74.7 |
| Central | 3 | −0.10 [−1.86, 1.65] | −0.25 | .827 | 0.46 (0.63) | 13.69 | .001 | 85.4 |
| Parietal | 2 | 0.02 [−6.42, 6.46] | 0.04 | .972 | 0.46 (0.73) | 8.60 | .003 | 88.4 |
| Temporal^a^ | 1 |  |  |  |  |  |  |  |
| Occipital | 3 | ­−0.37 [−2.24, 1.50] | −0.85 | .486 | 0.30 (0.53) | 4.88 | .087 | 59.0 |
| Relative Beta Power | | | | | | | | |
| Whole | 14 | 0.08 [−0.18, 0.33] | 0.64 | .535 | 0.11 (0.08) | 36.68 | .001 | 64.6 |
| Frontal | 5 | −0.17 [−0.83, 0.48] | −0.74 | .502 | 0.23 (0.24) | 19.74 | < .001 | 79.7 |
| Central | 3 | 0.30 [−0.43, 1.04] | 1.78 | .217 | 0.05 (0.15) | 2.38 | .304 | 16.0 |
| Parietal | 2 | 0.32 [−2.68, 3.32] | 1.35 | .405 | 0.06 (0.17) | 2.09 | .149 | 52.1 |
| Temporal^a^ | 1 |  |  |  |  |  |  |  |
| Occipital^a^ | 1 |  |  |  |  |  |  |  |
| Relative Gamma Power^c^ | | | | | | | | |
| Whole | 2 | **1.06 [0.65, 1.48]** | **32.52** | **.020** | 0.00 (0.22) | 0.01 | .904 |  |

*Note*. Primary meta-analytic models, indicated by “Whole” for topographical region, are included for ease of comparison. Significant (*p* < .05) and marginally significant (*p* < .10) pooled effect sizes are in bold and italics, respectively.

^a^ No supplemental meta-analytic models were fitted because only one study for a specific topographical region was available.

^b^ Supplemental meta-analytic models yielded null estimates.

^c^ No supplemental meta-analytic models were fitted for relative gamma power because no study for any specific topographical region was available.

**Supplementary Table 3**

*Sensitivity Analyses for Different Correlated Sampling Errors Used in Aggregation of Multiple, Within-Study Effect Sizes*

|  |  | Pooled Effect Size | | | Between-Study Heterogeneity | | | |
| --- | --- | --- | --- | --- | --- | --- | --- | --- |
| Correlation | *k* | Hedges’ *g* [95% CI] | *t* | *p* | τ^2^ (*SE*) | Cochran’s *Q* | *p* | *I*^2^ (%) |
| Absolute Delta Power | | | | | | | | |
| .1 | 15 | 0.06 [−0.23, 0.34] | 0.42 | .680 | 0.18 (0.09) | 93.67 | < .001 | 85.1 |
| .3 | 15 | 0.06 [−0.23, 0.34] | 0.42 | .679 | 0.19 (0.10) | 77.30 | < .001 | 81.9 |
| .5 | 15 | 0.06 [−0.23, 0.34] | 0.42 | .678 | 0.19 (0.10) | 68.98 | < .001 | 79.7 |
| Absolute Theta Power | | | | | | | | |
| .1 | 19 | −0.03 [−0.27, 0.20] | −0.29 | .773 | 0.16 (0.08) | 78.74 | < .001 | 77.1 |
| .3 | 19 | −0.03 [−0.27, 0.20] | −0.29 | .777 | 0.16 (0.08) | 72.76 | < .001 | 75.3 |
| .5 | 19 | −0.03 [−0.27, 0.20] | −0.28 | .782 | 0.16 (0.08) | 68.83 | < .001 | 73.8 |
| Absolute Alpha Power | | | | | | | | |
| .1 | 23 | *−0.17 [−0.37, 0.02]* | *−1.88* | *.073* | 0.12 (0.06) | 64.23 | < .001 | 65.7 |
| .3 | 23 | *−0.17 [−0.37, 0.02]* | *−1.89* | *.072* | 0.11 (0.06) | 57.56 | < .001 | 61.8 |
| .5 | 23 | *−0.18 [−0.37, 0.02]* | *−1.90* | *.070* | 0.11 (0.06) | 53.60 | < .001 | 59.0 |
| Absolute Beta Power | | | | | | | | |
| .1 | 16 | 0.01 [−0.13, 0.16] | 0.22 | .832 | 0.00 (0.01) | 25.82 | .040 | 41.9 |
| .3 | 16 | 0.01 [−0.13, 0.15] | 0.17 | .868 | 0.00 (0.01) | 23.47 | .075 | 36.1 |
| .5 | 16 | 0.01 [−0.13, 0.16] | 0.16 | .877 | 0.00 (0.01) | 22.23 | .102 | 32.5 |
| Absolute Gamma Power | | | | | | | | |
| .1 | 9 | **0.37 [0.00, 0.73]** | **2.31** | **.050** | 0.12 (0.09) | 21.24 | .007 | 62.3 |
| .3 | 9 | **0.37 [0.00, 0.75]** | **2.32** | **.049** | 0.12 (0.10) | 20.85 | .008 | 61.6 |
| .5 | 9 | **0.38 [0.00, 0.76]** | **2.32** | **.049** | 0.13 (0.10) | 20.59 | .008 | 61.1 |
| Relative Delta Power | | | | | | | | |
| .1 | 12 | 0.10 [−0.26, 0.45] | 0.60 | .558 | 0.23 (0.13) | 85.54 | < .001 | 87.1 |
| .3 | 12 | 0.10 [−0.26, 0.45] | 0.60 | .563 | 0.23 (0.13) | 70.63 | < .001 | 84.4 |
| .5 | 12 | 0.10 [−0.26, 0.45] | 0.59 | .568 | 0.22 (0.13) | 61.96 | < .001 | 82.2 |
| Relative Theta Power | | | | | | | | |
| .1 | 14 | −0.17 [−0.67, 0.32] | −0.76 | .462 | 0.55 (0.25) | 75.39 | < .001 | 82.8 |
| .3 | 14 | −0.15 [−0.62, 0.33] | −0.67 | .515 | 0.45 (0.22) | 66.18 | < .001 | 80.4 |
| .5 | 14 | −0.12 [−0.57, 0.33] | −0.58 | .572 | 0.38 (0.19) | 61.15 | < .001 | 78.7 |
| Relative Alpha Power | | | | | | | | |
| .1 | 17 | **−0.34 [−0.61, −0.08]** | **−2.76** | **.014** | 0.17 (0.09) | 96.54 | < .001 | 83.4 |
| .3 | 17 | **−0.35 [−0.61, −0.08]** | **−2.80** | **.013** | 0.17 (0.09) | 83.45 | < .001 | 80.8 |
| .5 | 17 | **−0.35 [−0.61, −0.09]** | **−2.82** | **.012** | 0.17 (0.09) | 74.75 | < .001 | 78.6 |
| Relative Beta Power | | | | | | | | |
| .1 | 14 | 0.07 [−0.18, 0.33] | 0.61 | .552 | 0.11 (0.08) | 40.09 | < .001 | 67.6 |
| .3 | 14 | 0.08 [−0.18, 0.33] | 0.64 | .535 | 0.11 (0.08) | 36.68 | .001 | 64.6 |
| .5 | 14 | 0.08 [−0.18, 0.33] | 0.65 | .524 | 0.11 (0.08) | 34.53 | .001 | 62.4 |
| Relative Gamma Power | | | | | | | | |
| .1 | 2 | **1.06 [0.65, 1.48]** | **32.52** | **.020** | 0.00 (0.22) | 0.01 | .904 |  |
| .3 | 2 | **1.06 [0.65, 1.48]** | **32.52** | **.020** | 0.00 (0.22) | 0.01 | .904 |  |
| .5 | 2 | **1.06 [0.65, 1.48]** | **32.52** | **.020** | 0.00 (0.22) | 0.01 | .904 |  |

*Note*. Primary meta-analytic models assumed a medium-sized correlation of .3 in the compound symmetric variance-covariance structure. Sensitivity analyses were conducted with a small-sized correlation of .1 and a large-sized correlation of .5. Significant (*p* < .05) and marginally significant (*p* < .10) pooled effect sizes are in bold and italics, respectively.

**Supplementary Figure 1**

*Contour-Enhanced Funnel Plots for Absolute EEG Power Differences*


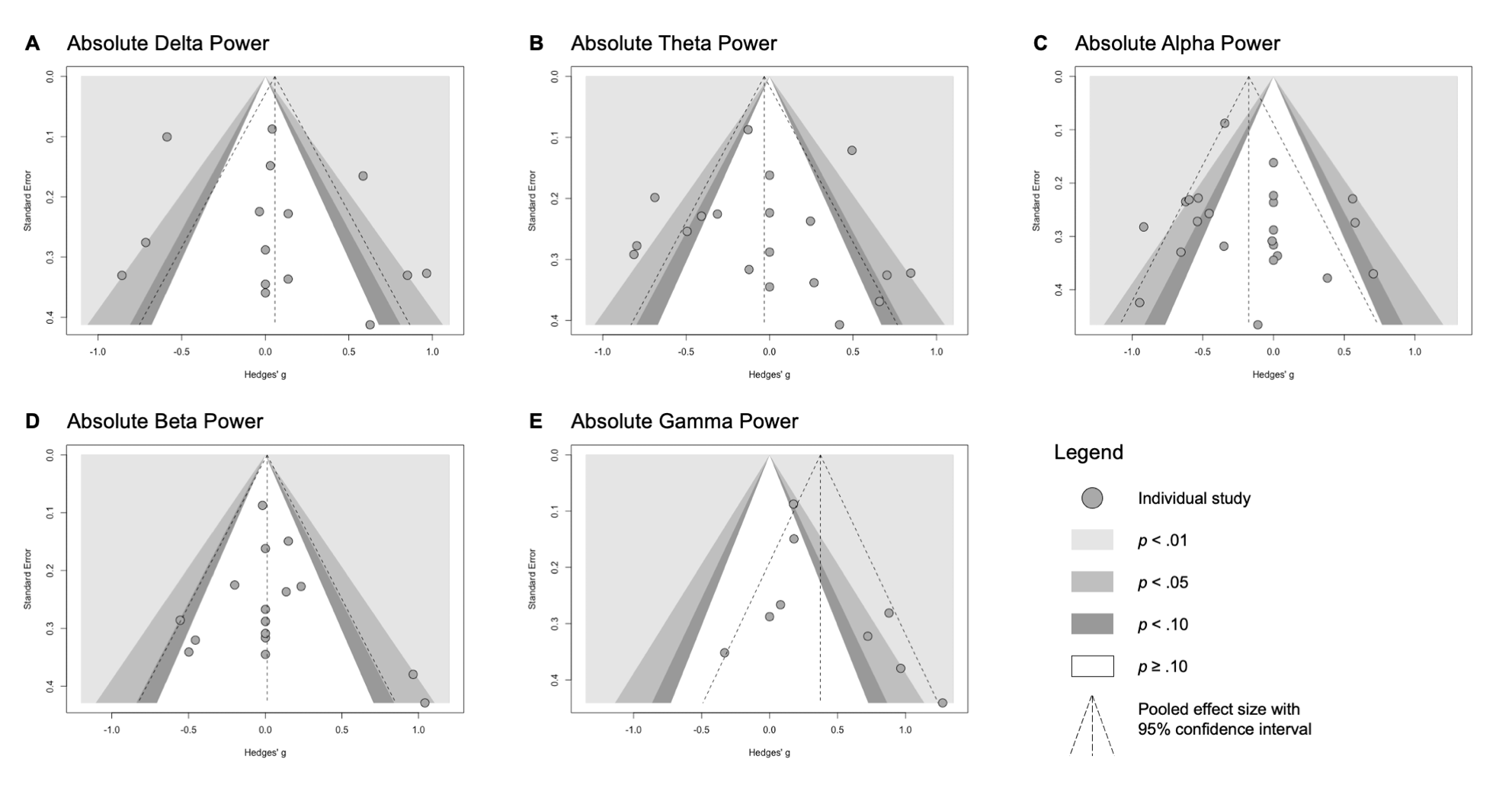


*Note*. Contour-enhanced funnel plots are depicted separately for absolute (A) delta, (B) theta, (C) alpha, (D) beta, and (E) gamma power differences between autistic and neurotypical individuals. Positive effect sizes indicate greater absolute power in autistic individuals than neurotypical individuals.

**Supplementary Figure 2**

*Contour-Enhanced Funnel Plots for Relative EEG Power Differences*


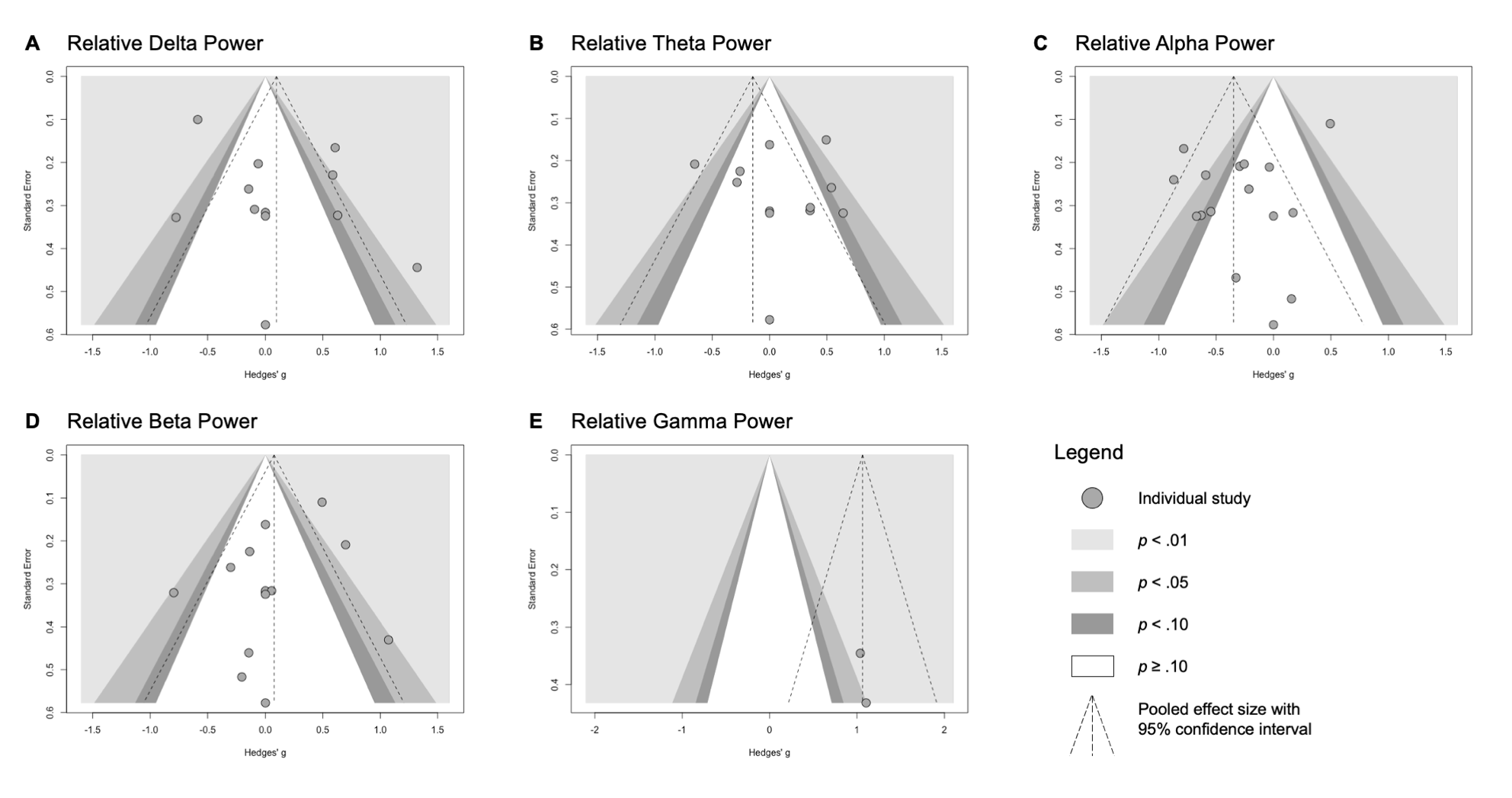


*Note*. Contour-enhanced funnel plots are depicted separately for relative (A) delta, (B) theta, (C) alpha, (D) beta, and (E) gamma power differences between autistic and neurotypical individuals. Positive effect sizes indicate greater relative power in autistic individuals than neurotypical individuals.
